# “Iron free” zinc oxide nanoparticles with ion-leaking properties disrupt intracellular ROS and iron homeostasis to induce ferroptosis

**DOI:** 10.1038/s41419-020-2384-5

**Published:** 2020-03-13

**Authors:** Changping Zhang, Zixuan Liu, Yuhao Zhang, Liang Ma, Erqun Song, Yang Song

**Affiliations:** 1 Key Laboratory of Luminescence and Real-Time Analytical Chemistry (Southwest University), Ministry of Education, College of Food Science, Southwest University, Chongqing, 400715 People’s Republic of China; 2 Key Laboratory of Luminescence and Real-Time Analytical Chemistry (Southwest University), Ministry of Education, College of Pharmaceutical Sciences, Southwest University, Chongqing, 400715 People’s Republic of China

**Keywords:** Biophysical chemistry, Stress signalling

## Abstract

Exposure to nanomaterials (NMs) is an emerging threat to human health, and the understanding of their intracellular behavior and related toxic effects is urgently needed. Ferroptosis is a newly discovered, iron-mediated cell death that is distinctive from apoptosis or other cell-death pathways. No evidence currently exists for the effect of “iron free” engineered NMs on ferroptosis. We showed by several approaches that (1) zinc oxide nanoparticles (ZnO NPs)-induced cell death involves ferroptosis; (2) ZnO NPs-triggered ferroptosis is associated with elevation of reactive oxygen species (ROS) and lipid peroxidation, along with depletion of glutathione (GSH) and downregulation of glutathione peroxidase 4 (GPx4); (3) ZnO NPs disrupt intracellular iron homeostasis by orchestrating iron uptake, storage and export; (4) p53 largely participates in ZnO NPs-induced ferroptosis; and (5) ZnO particle remnants and dissolved zinc ion both contribute to ferroptosis. In conclusion, our data provide a new mechanistic rationale for ferroptosis as a novel cell-death phenotype induced by engineered NMs.

## Introduction

Nanomaterials (NMs) readily enter the human body through respiratory inhalation, oral ingestion or a dermal route, cross the plasma membrane and reach the inside of cells, ultimately triggering cell death^1^. The disruption of cell-death homeostasis is associated with various diseases, including neurodegenerative diseases, immune disorders, diabetes and cancers. Thereafter, it is important to elaborate the molecular processes of NMs-regulated cell death^2,3^. Among them, the majority of cell-death modalities are associated with NMs-induced cytotoxicities.

Ferroptosis is a recently recognized cell death with unique morphological, genetic and biochemical characteristics that are distinct from apoptosis, autophagy or necrosis^4,5^. Ferroptosis is an iron-dependent accumulation of lipid reactive oxygen species (ROS) process. Small molecule inducers or inhibitors of ferroptosis through targeting iron metabolism or lipid peroxidation have been extensively studied^6^.

Advances in nanotechnology have stimulated the enthusiasm in the designing of multifunctional NMs for cancerous therapeutic applications through ferroptotic mechanisms^7^. However, most of the studies used iron or iron-oxide-based NMs^8–10^. Nevertheless, in addition to biomedical applications, human beings are unconsciously exposed to numerous of engineered NMs through consumer products. To this end, from a safety perspective, we ought to assess whether ferroptosis occurs upon “iron free” engineered NMs exposure and decipher the corresponding mechanism(s).

ZnO nanoparticles (ZnO NPs) are often used in food additives for nutritional purposes, sunscreen cream for absorbance of UV light, and antimicrobial agents for skin protection. In addition, ZnO NPs have been applied to many medical applications, such as drug delivery, tissue regeneration and bioimaging^11^. Although ZnO NPs are listed as safe materials by the United States Food and Drug Administration (USFDA), they are one of the most toxic metallic oxide nanoparticles^11^. Using ZnO NPs as a model, we demonstrated that ferroptosis is a novel form of cell death induced by NMs. Moreover, the possibilities of other “iron free” NMs (metal oxide, carbon, gold, and silver-based) on ferroptosis are also discussed.

## Materials and methods

### Chemical and reagents

Cell Counting Kit-8 (CCK8) assay kit was purchased from Bimake Inc. (USA). 2′,7′-dichlorofluorescein diacetate (DCFH-DA) was purchased from Sigma-Aldrich Inc. (Sigma, USA). MitoTracker® Deep Red FM, Lyso Tracker Red, 1,1′-dioctadecyl-3,3,3′,3′-tetramethylindotricarbocyanine perchlorate (DiI), JC-1 staining kit and propidium iodide (PI) were purchased from YE SEN Inc. (Shanghai, China). N-(6-methoxy-8-quinolyl)-*p*-toluenesul fonamide (TSQ) was purchased from AAT BIOQUEST (USA). Annexin V-FITC/PI, lactate dehydrogenase (LDH), the cleaved caspase 8 and caspase 3 polyclonal primary antibodies from Wanleibio (Nanjing, China). Malondialdehyde (MDA), glutathione (GSH), and Glutathione peroxidase (GPx) assay kit were purchased from Nanjing Jiancheng Bioengineering Institute (Nanjing, China). Acridine orange-ethidium bromide (AO-EB) double stain kit and Lillie divalent iron staining solution were supplied by Solarbio Inc. (Beijing, China). Ferrostatin-1 (Fer-1), desferrioxamine (DFO), Liproxstatin-1 (LIP-1), (E)-Necrosulfonamide (NSA), chloroquine (CQ), and Z-VAD-FMK were supplied by MedChemExpress (Shanghai, China). Necrostatin-1 (Nec-1) and 3-methyladenine (3-MA) were purchased from Target Mol (Boston, USA). The mouse p53, ferritin heteropolymers ferritin light chain (FTL) and ferritin heavy chain (FTH), iron importers transferrin receptor protein 1 (TFRC), SLC7A11, and GPx4 polyclonal primary antibodies were purchased from Santa Cruz Biotechnology (USA). The mouse SAT1 and DMT1 polyclonal primary antibodies were purchased from Bioss (Beijing, China). β-Actin polyclonal primary antibody, goat-anti-rabbit IgG-HRP conjugated secondary antibody, and goat-anti-mouse IgG-HRP conjugated secondary antibody were supplied by Sangon Biotech Co, Ltd. (Shanghai, China). ZnO NPs (>99.9% purity) and ZnCl_2_ standard solution (0.1 M) were purchased from Aladdin Reagent Database Inc. (Shanghai, China).

### Fluorescent labeling of ZnO NPs

Briefly, 20 mg ZnO NPs were dispersed in 15 mL absolute ethanol. A solution of 30 μL APTS diluted in 1 mL absolute ethanol was added to the particle suspensions, sonicated and stirred under nitrogen atmosphere at room temperature for 20 h. The modified NPs were collected by centrifuging and removing the supernatant. After washing, the modified NPs were resuspended in 15 mL absolute ethanol and mixed with a solution contain 1 mg FITC and 0.5 mL absolute ethanol. The suspension was stirred for 4 h and fluorescein isothiocyanate (FITC)-labeled (F-ZnO) NPs were collected by centrifugation. After thorough washing of the labeled materials with absolute ethanol, the particles were dried under vacuum to remove the organic solvent and stored as dry powders. The maximum absorption peak of synthesized F-ZnO NPs is 539 nm.

### Characterization of ZnO and F-ZnO NPs

ZnO NPs and F-ZnO NPs were characterized by TEM (JEM1200EX, Japan). The sample was diluted with water, dropped on a carbon film copper mesh, and the sample was dried and stained with uranyl acetate, and naturally dried in a fume hood. The dried sample was placed in an observation room with an accelerating voltage of 120 kV and photographed. The crystalline nature of ZnO NPs was carried out by XRD (BRUCKER D8, Germany). Place the sample into the sample stage and compact it into a flat surface. The tube current is 40 mA, the tube voltage is 40 kV, the Cu target wavelength is 1.5406 Å, and the Co target wavelength is 1.75926 Å. The X-ray patterns were matched literature. Hydrodynamic diameter, PDI and zeta potential of ZnO and F-ZnO NPs were determined using a Mastersizer Micro (Malvern, UK) in pure water and RPMI1640 medium. ZnO NPs and F-ZnO NPs were dispersed in water at a concentration of 15 µg/mL, respectively. Then, the suspensions were sonicated using a sonicator bath at room temperature for 10 min at 80 W.

### Cell culture

HUVECs, LO2, and RAW264.7 obtained from the American Type Culture Collection (ATCC, USA), cultured in complete RPMI1640 medium containing 10% fetal bovine serum (FBS). MDA-MB231, Hepa 1-6, and Hela obtained from the American Type Culture Collection (ATCC, USA), cultured in complete DMEM medium containing 10% fetal bovine serum (FBS). PC12, obtained from the American Type Culture Collection (ATCC, USA), was cultured in complete RPMI1640 medium containing 10% newborn calf serum (NCS). MDA-MB-453 was cultured in complete L15 medium containing 10% NCS. BT-474, obtained from cell bank of the Chinese Academy of Sciences, was cultured in complete RPMI1640 medium containing 15% FBS. Cells were incubated at 37 °C in a humidified atmosphere of 5% CO_2_ in air.

### Cell viability assay

Cell viability was determined by CCK8 assay. HUVECs (5 × 10^4^/well) were seeded in a 96-well plate overnight before treatment with ZnO NPs for 24 h with or without other co-treatment. Subsequently, 100 μL of CCK8 solution (10%) was added to each well and incubated at 37 °C for 2 h. The absorbance of each culture well was measured with a microplate reader (BioTek, USA) at a wavelength of 450 nm.

### LDH release assay

Briefly, cells were cultured in 24-well plates with ~8 × 10^4^ cells per well. After 12 h of growth, cells were then treated with ZnO NPs at concentrations of 0, 5, 10, and 15 μg/mL for 24 h. The supernatants were collected, 100 μL cell medium was used for LDH activity analysis, the absorbance at 450 nm was measured by microplate reader (BioTek ELX800, USA). The amount of LDH released is expressed as LDH activity (U/L) in culture media.

### Annexin V-FITC/PI staining analysis

Annexin V-FITC/PI staining was performed on a BD FACS Melody^TM^ flow cytometry. After 24 h of growth, cells were treated by ZnO NPs at concentrations of 0, 5, 10, and 15 μg/mL for 24 h. In each group, at least 1 × 10^4^ cells were analyzed to determine the percentage of apoptotic cells. The results are presented as percentage of apoptotic cells (including early and late apoptotic cells).

### PI staining analysis

Approximately 1 × 10^6^ cells/well were seeded in 6-well plates overnight followed by the treatment of ZnO NPs for 24 h. Corresponding inhibitors or activators were introduced 1 h prior to ZnO NPs (10 μg/mL) treatment. Cells were incubated with PI staining in the dark at 37 °C for 15 min. Cells were washed twice with PBS. The cellular fluorescence was analyzed by a BD FACS Melody^TM^ flow cytometry. A total of 1 × 10^4^ event was acquired for each sample from three independent experiments.

### AO-EB double staining

Cells were cultured in confocal cell culture dish with ~8 × 10^4^ cells per well. After 24 h of growth, cells were treated by ZnO NPs at concentrations of 0, 5, 10, and 15 μg/mL for 24 h. Then, cells were washed twice with PBS; then, they were incubated with AO-EB staining in the dark at 37 °C for 5 min. After the stained cells were rinsed three times with PBS, the cells were examined under a reversed fluorescence microscope (Olympus IX71).

### Mitochondrial membrane potential (ΔΨm, MMP) assay

Cells were cultured in confocal cell culture dish with ~8 × 10^4^ cells per well. After 24 h of growth, cells were treated with ZnO NPs at concentrations of 0, 5, 10, and 15 μg/mL for 24 h. The changes in ΔΨ_m_ were monitored after staining with JC-1 (1 μg/mL) probe. Analysis was performed on a BD FACS Melody^TM^ flow cytometry.

### Mitochondrial morphology analysis

Cells were cultured in confocal cell culture dish, with ~8 × 10^4^ cells per well. After 24 h of growth, cells were treated by ZnO NPs at concentrations of 10 μg/mL for additional 24 h. Erastin (25 μM) was used as a positive control. Cells were washed twice with PBS, then, cells were incubated with Mito Tracker® Deep Red FM in the dark at 37 °C for 30 min. After the stained cells were rinsed three times with PBS, cells were imaged under confocal microscope (Nikon N-SIM).

### TEM analysis of cells

TEM analysis was performed to observe the morphology and microstructure of ZnO NPs-treated HUVECs. Briefly, HUVECs were treated with ZnO NPs (10 μg/mL) or erastin (25 μM) for 24 h. Before cells collection, cells were washed twice with PBS and fixed in 4% glutaraldehyde at room temperature for 1 h followed by 4 °C for additional 12 h. Next, cells were post-fixed with 1% OsO_4_ and embedded in epon. Then, ultrathin sections were stained with uranyl acetate/lead citrate and visualized in a Hitachi-7500 TEM instrument (Hitachi, Tokyo, Japan).

### Lillie divalent iron staining

Cells were cultured in confocal cell culture dish, with ~8 × 10^4^ cells per well. After 24 h of growth, cells were treated by NPs or ZnCl_2_ for additional 24 h. After washed twice with PBS, cells were incubated with Lillie divalent iron staining reagent in the dark at 37 °C for 1 h. After rinsed three times with PBS, the cells were examined under a reversed fluorescence microscope (Olympus IX71).

### ROS level measurement

Approximately 1 × 10^6^ cells/well were seeded in 6-well plates overnight followed by the treatment of ZnO NPs for 24 h. Corresponding inhibitors or activators were introduced 1 h prior to ZnO NPs treatment. Cells were incubated with DCFH-DA indicator in the dark at 37 °C for 30 min. Cells were washed twice with PBS. The cellular fluorescence was analyzed by a BD FACS Melody^TM^ flow cytometry. A total of 1 × 10^4^ events were acquired for each sample from three independent experiments. Data were analyzed by FlowJo V10.

### GSH level measurement

HUVECs at a concentration of 2 × 10^5^ cells/well were seeded in 6-well plates. Cells were treated with ZnO NPs (0, 5, and 10 μg/mL) for 24 h. Cells were harvested and cell number were determined. Total GSH was measured by a commercial GSH kit according to the manufacturer’s instructions.

### MDA level measurement

HUVECs at a concentration of 2 × 10^5^ cells/well were seeded in 6-well plates. Cells were treated as ZnO NPs (0, 5, and 10 μg/mL) for 24 h. Cells were harvested and cell numbers were determined. MDA level was measured by a commercial MDA kit according to the manufacturer’s instructions.

### siRNA interference

HUVECs were transfected with 25 nM siRNA for P53, SAT1, ACSL4, ALOX15 or scrambled siRNA using siRNA-mate transfection reagent according to the manufacturer’s protocol. The sense-strand sequences of siRNA duplexes were listed in Supplementary Table 3.

### Western blotting assay

HUVECs at a concentration of 2 × 10^5^ cells/well were seeded in 6-well plates. Cells were treated with ZnO NPs (0, 5, and 10 μg/mL) for 24 h. After harvest, the protein concentrations were detected by the BCA protein assay according to the manufacturer’s instruction. Thirty micrograms of cellular protein from each group was blotted onto PVDF membrane following separation on 12.5% SDS-PAGE. The immuno-blot was incubated with the blocking solution (8% skim milk) at room temperature for 2 h, followed by incubation with a corresponding primary antibody at 37 °C for 3 h. After washing with Tween 20/Tris-buffered saline (TBST), the immune-blot was incubated with respective secondary antibody (1:5000) for 2 h at room temperature. Followed by visualization using the ECL system. Representative images were chosen from at least three independent experiments. Protein expression levels were standardized by β-actin in different cell substrates.

### RNA extraction and real-time quantitative PCR (RT-qPCR)

Total RNA was extracted with a total RNA purification kit (BioTeke, Beijing, China) as described in the manufacturer’s instructions. The purified RNA (2 μg) was reverse transcribed into cDNA with the transcriptor first strand cDNA synthesis kit (Roche, Switzerland). Then, cDNA was used to perform RT-qPCR analysis using the Light Cycler 96 instrument protocol with FastStart Essential DNA Green Master (Roche, Switzerland). Subsequently, 35 cycles of PCR were carried out with denaturation at 94 °C for 20 s, annealing at the most suitable temperature for 30 s and extension at 72 °C for 30 s, followed by a final incubation at 72 °C for 10 min. The primers used for the amplification were listed in Supplementary Table 4.

### Atomic absorption spectroscopy (AAS) assay

HUVECs were seeded in 10 cm plates and exposed to ZnO NPs or ZnCl_2_ dispersions for 24 h. Then, cells were thoroughly washed, harvested, and counted. The supernatant and total Zn^2+^ concentration in solution was determined using AAS in the graphite furnace mode (TAS-990, Persee, China). For dissociated Zn^2+^ concentration measurement, the cell lysate was centrifuged at 30,000 × *g* for 1 h and supernatant was collected. For total Zn^2+^ concentration measurement, the cell lysate was acidified to pH < 2 with 65% HNO_3_, followed by digestion (1 mL sample + 1 mL 30 wt% H_2_O_2_, 3 mL 65 wt% HNO_3_ via water bath 75 °C for 6 h). Each experiment was performed in triplicate.

### Zn^2+^ release in PBS and ALF

The Zn^2+^ release was also measured in PBS or ALF (artificial lysosomal fluid). The PBS (pH of 7.4) was used to mimic the physiological environment. The ALF (pH of 4.5) was intended to mimic the lysosomal acidic environment. ALF composition is according to previous publication^12^. ZnO NPs dispersions were prepared in PBS or ALF and kept at 37 °C. After 24 h samples were centrifuged for 1 h at 15,000 × *g*, 4 °C, the supernatant was collected and analyzed by AAS.

### Intracellular zinc imaging

HUVECs was pretreated in a culture medium containing 10 μg/mL of F-ZnO NPs for 24 h. After permeabilization and fixation, cells were then rinsed three times with PBS and stained in a PBS solution contains cellular membrane marker DiI (20 μM) or Zn^2+^-specific fluorescent dye TSQ (30 μM) for 30 min. The cells were examined under a super-resolution confocal microscope (Nikon N-SIM) with an excitation wavelength of 405 nm. The average fluorescence intensity was quantitatively measured to reflect the intracellular Zn^2+^ concentration in HUVECs. The lysosomes were stained by 1 μM Lyso Tracker Red (YE SEN, China) and visualized with an excitation wavelength of 635 nm for the determination of subcellular location of ZnO NPs. F-ZnO NPs was visualized at an excitation wavelength of 488 nm.

### Statistical analysis

All statistical analyses were evaluated by a one-way ANOVA and followed by a Tukey’s multiple-comparisons test. *P* < 0.05 was considered statistically significant. All the dates were expressed as mean ± standard deviations (S.D.).

## Results

### Characterization of ZnO and fluorescein isothiocyanate (FITC)-labeled (F-ZnO) NPs

The morphology and average size of ZnO NPs were determined by transmission electron microscopy (TEM). ZnO NPs have a near-spherical shape with an average primary diameter of 30 ± 10 nm (Supplementary Fig. 1). ZnO NPs characterized by X-ray diffraction (XRD) with CuKα radiation revealed a crystalline nature structure which is consistent with the standard zincite, JCPDS 5-0664 (Supplementary Fig. 2). The hydrodynamic sizes and zeta potentials of ZnO and F-ZnO NPs were measured in ultrapure water and RPMI1640 medium after incubation at 37 °C (Supplementary Table 1). Both ZnO and F-ZnO NPs have a relatively uniform size with a low polydispersity index (PDI).

### Cytotoxicity of ZnO NPs on human umbilical vein endothelial cells (HUVECs)

Epidemiologic and experimental studies have both demonstrated a correlation between NMs exposure and an increased incidence of cardiovascular diseases^13,14^. The current study was mainly conducted in HUVECs due to their cardiovascular relevance. ZnO NPs caused obvious decreases in cell viability (*P* < 0.01 in the 10 μg/mL group and *P* < 0.001 in the 15 μg/mL group) (Supplementary Fig. 3). A dose-dependent increase in LDH release after ZnO NPs treatment suggested ZnO NPs-induced cell death (Supplementary Fig. 4). Then, annexin V-FITC/propidium iodide (PI) staining results revealed a dose-dependent increase in annexin V-FITC/PI-positive cells after ZnO NPs challenge (~39% in the 15 μg/mL group) (Supplementary Fig. 5). An acridine orange-ethidium bromide (AO-EB) staining assay revealed reduced green fluorescence and an increased orange fluorescence, suggesting necrotic-like cell death (Supplementary Fig. 6). Moreover, mitochondrial damage after exposure to ZnO NPs further confirmed ZnO NPs-associated cytotoxicity in HUVECs (Supplementary Fig. 7). Consistently, we also observed the cleavage of caspase 8 and 3, which are hallmarks of apoptosis activation parthanatos (Supplementary Fig. 8). For the following experiments, the maximum concentration of ZnO NPs exposure was set as 10 μg/mL (except for Zn^2+^ concentration measurement) to maintain >80% viable cells.

### ZnO NPs trigger oxidative stress-dependent, iron-mediated ferroptosis

**Fig. 1:**
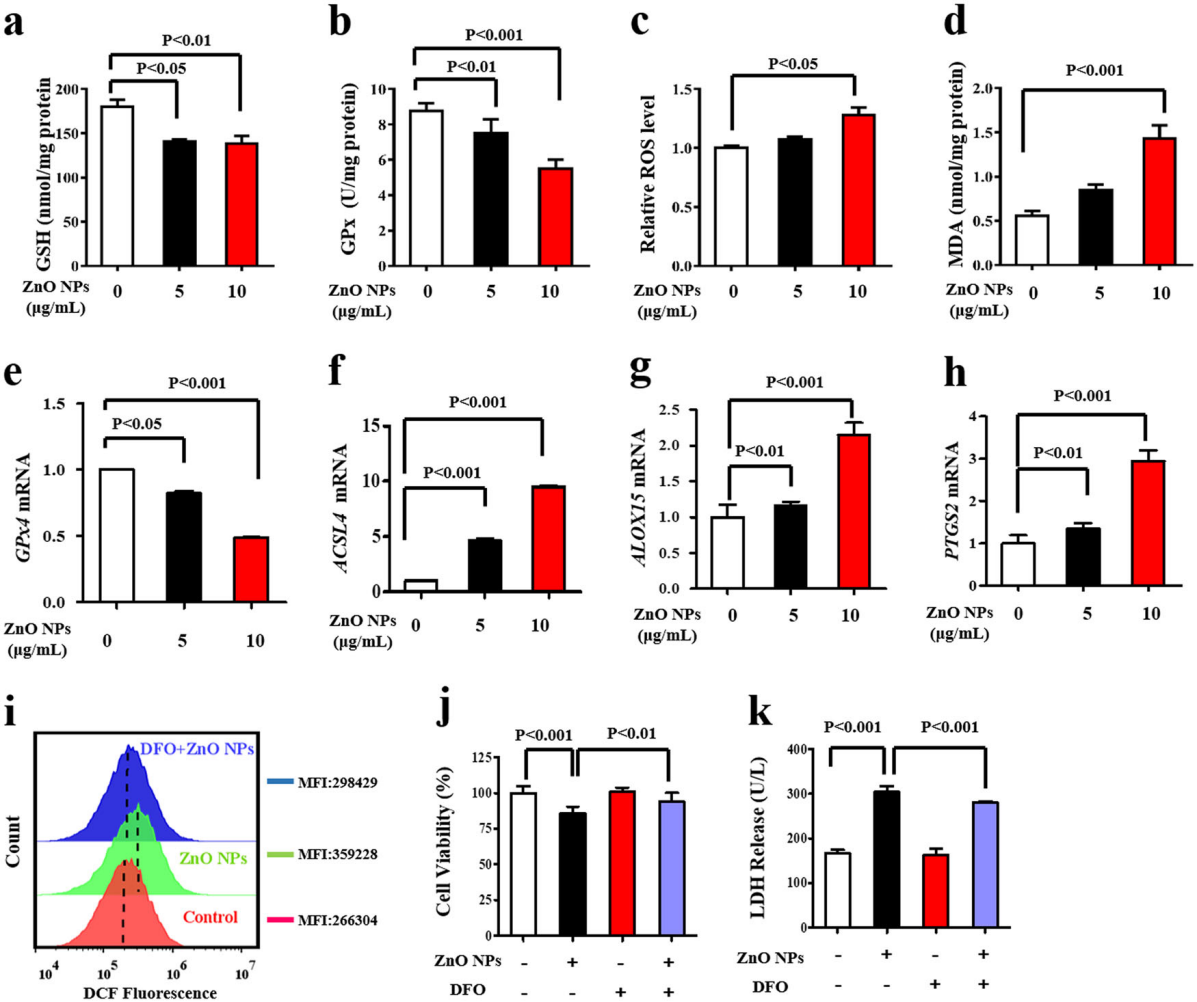
ZnO NPs trigger oxidative stress-dependent, iron-mediated ferroptosis. HUVECs were treated with ZnO NPs (0, 5, and 10 μg/mL) for 24 h. **a** GSH, **b** GPx, **c** ROS, and **d** MDA levels were measured. Total RNA was extracted for the analysis of mRNA levels of interest using qRT-PCR. **e** GPx4, **f** ACSL4, **g** ALOX15, and **h** PTGS2 mRNA. HUVECs were pretreated with DFO (100 μM) for 1 h in the presence of 10 μg/mL ZnO NPs. **i** The generation of ROS was determined by the DCFH-DA probe, MFI: mean fluorescence intensity. **j** Cell viability analysis. **k** LDH leakage assay. Data are shown as the mean ± S.D. from three independent experiments.

ROS generation and antioxidant depletion are common in ZnO NPs-treated cells^15^. Our results established that ZnO NPs cause glutathione (GSH) (Fig. 1a) and glutathione peroxidase (GPx) depletion (Fig. 1b). Using the DFCH-DA probe as an ROS indicator, the results showed that ZnO NPs increase ROS levels in a dose-dependent manner (Fig. 1c). Consistently, malondialdehyde (MDA) levels were dramatically increased (Fig. 1d). Together, these results indicated ROS elevation caused by ZnO NPs.

Previous studies have described that not only apoptosis, but also necrosis and autophagy were induced by ZnO NPs^11,16,17^. Here, we questioned the involvement of ferroptosis in ZnO NPs-mediated cell death. We observed that the addition of specific cell-death inhibitors decreased the relative PI fluorescence induced by ZnO NPs (10 μg/mL) (Supplementary Fig. 9). The rescue effects of various inhibitors are consistent with previous studies that showed ZnO NPs-induced apoptosis, necroptosis, and autophagy^16,17^. Ferroptosis has its distinctive characteristics^6,18^, we further evaluated whether ZnO NPs lead to lipid peroxidation by measuring GPx4 levels at the transcriptional and posttranscriptional levels. Indeed, ZnO NPs decreased GPx4 mRNA (Fig. 1e) and protein levels (Supplementary Fig. 10), respectively. Together, the inhibition of system X_c_^−^ and promotion of lipid peroxidation are critical events of ZnO NPs exposure, implying the occurrence of ferroptosis. Acyl-CoA synthetase long-chain family member 4 (ACSL4) contributes to the accumulation of lipid intermediates during ferroptosis and the loss of ACSL4 gene resulted in ferroptosis resistance^19^. Alternatively, ferroptosis is promoted by lipoxygenase (LOX)-catalyzed lipid hydroperoxide generation^20^. Therefore, we analyzed ACSL4 and ALOX15 genes by qRT-PCR, and the results indicated upregulation of expression of both genes (Fig. 1f, g). Furthermore, silencing of ACSL4 and ALOX15 decreased ZnO NPs-induced elevation of DCF fluorescence intensity, indicated the inhibition of intracellular ROS (Supplementary Fig. 11). The upregulation of prostaglandin-endoperoxide synthase 2 (PTGS2) has recently been identified as a potential molecular marker of ferroptosis^18^. In accordance, PTGS2 was found to be remarkably upregulated when HUVECs were treated with ZnO NPs (Fig. 1h).

Next, we revealed whether ferrous iron was required for ZnO NPs’ action through the co-treatment of cells with the potent iron chelator desferrioxamine (DFO). ZnO NPs-increased ROS level was almost completed attenuated in the presence of DFO (Fig. 1i). The co-treatment of DFO prevented cell death and LDH release triggered by ZnO NPs (Fig. 1j, k). The occurrence of ferroptosis in HUVECs line was confirmed by erastin (Supplementary Fig. 12). The combination of these results provides solid evidence of the ZnO NPs-induced susceptibility of HUVECs to ferroptosis.

### ZnO NPs exposure disrupts iron homeostasis

**Fig. 2:**
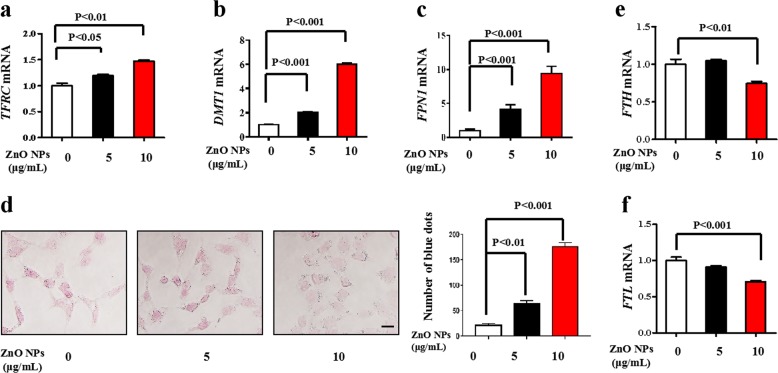
ZnO NPs exposure disrupts iron homeostasis. HUVECs were treated with ZnO NPs (5 or 10 μg/mL) for 24 h and total RNA was extracted for the analysis of **a** TFRC, **b** DMT1, and **c** FPN1 mRNA levels. **d** Lillie divalent iron staining. Scale bar = 20 μm. **e** FTH and **f** FTL mRNA levels. Data are shown as the mean ± S.D. from three independent experiments.

Since the iron chelator DFO rescues ZnO NPs-induced cell death, we further studied the effect of ZnO NPs on iron homeostasis. Cellular iron homeostasis is orchestrated perfectly through three processes, i.e., uptake, storage and export^6^. As shown, the mRNA levels of iron importers transferrin receptor protein 1 (TFRC) (mediates iron import) and divalent metal transporter 1 (DMT1) [facilitates Fe^2+^ transport to a labile iron pool (LIP) in the cytoplasm] were both significantly upregulated (Fig. 2a, b). Iron export is controlled solely by the iron efflux pump ferroportin (FPN1)^21^. In addition to increasing iron uptake, ZnO NPs increase intracellular iron by modulating the iron-export gene levels. Therefore, systemic iron homeostasis is governed by the hepcidin-ferroportin signaling axis. We discovered a noticeable increase in FPN1 at the transcriptional level by ZnO NPs (Fig. 2c). FPN1 can be regulated at the transcriptional, posttranscriptional and posttranslational levels^22,23^. Hepcidin, an iron-regulatory hormone, posttranslationally regulates FPN1 through its binding and proteolysis of FPN1 in lysosomes^24^. However, contradictory effects were obtained, e.g., increased Bach1 and MZF1 mRNA levels upon ZnO NPs exposure (Supplementary Fig. 13).

A direct measurement of free iron in the cellular compartment is necessary for reveal the effect of ZnO NPs on iron homeostasis. A commonly used calcein-AM assay for LIP measurement is not suitable in our study due to the effect of Zn^2+^ released by ZnO NPs. We thus investigated ferrous iron accumulation by Lillie ferrous iron staining assay. The obvious increases in deep blue *foci* in the ZnO NPs groups are evidence of an increase in ferrous iron (Fig. 2d). Consistently, the mRNA levels of FTH and FTL were significantly downregulated by ZnO NPs (Fig. 2e, f). Consistently, the iron inbound protein TFRC and DMT1 expressions were upregulated, and the iron outbound protein FTH and FTL expressions were downregulated (Supplementary Fig. 14). Undoubtedly, these results suggested defects in mitochondrial iron transport and utilization that suggested mitochondrial iron overload^25^.

### ZnO NPs exposure dysregulates mitochondrial dynamics

**Fig. 3:**
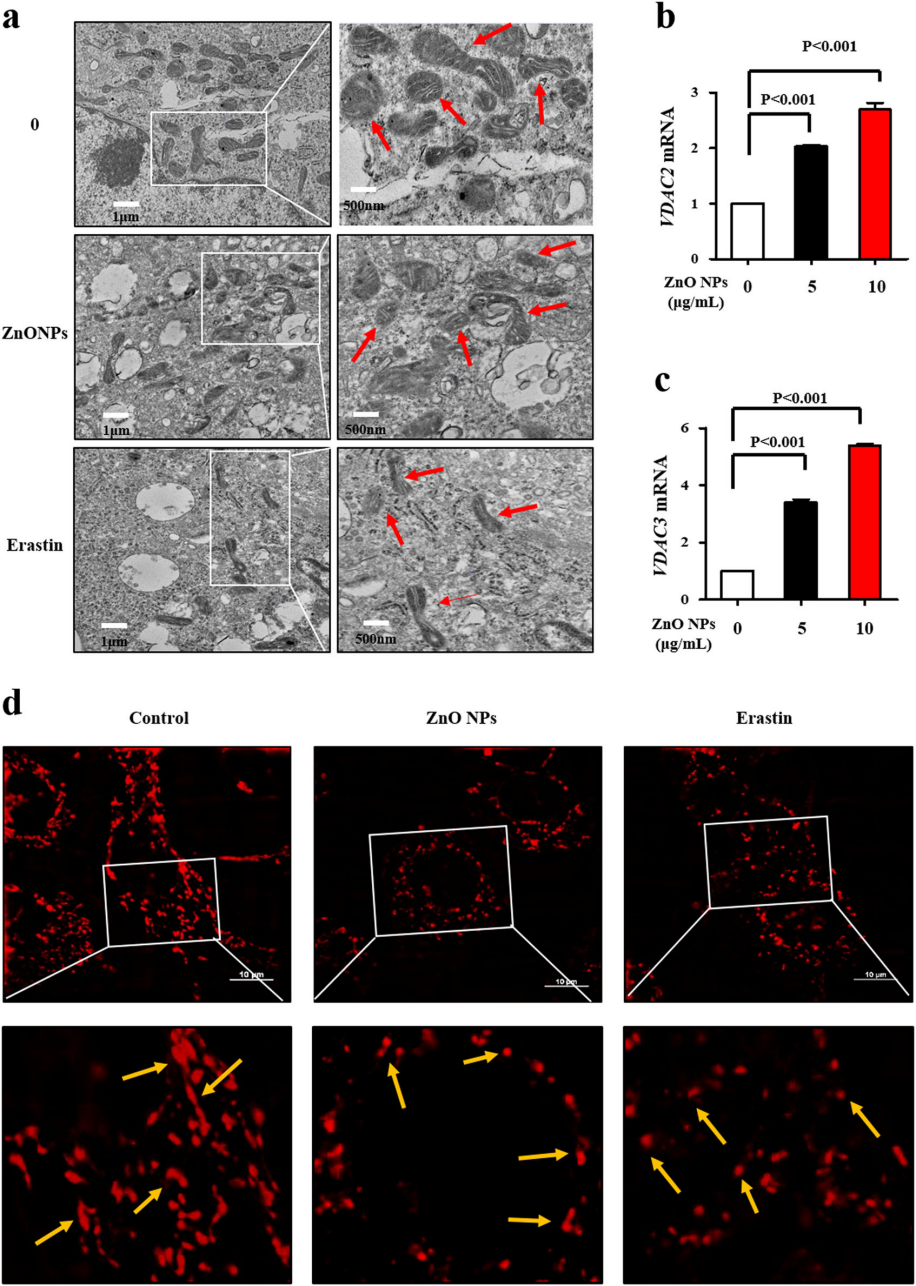
ZnO NPs exposure dysregulates mitochondrial dynamics. HUVECs were treated with ZnO NPs (10 μg/mL) or erastin (25 μM) for 24 h. **a** Typical TEM morphological images of ZnO NPs or erastin-treated cells. Scale bar = 1 μm. Total RNA was extracted for the analysis of (**b**) VDAC2 and (**c**) VDAC3 mRNA levels using qRT-PCR. **d** Representative MitoTracker ® Deep Red FM staining (50 nM) to assess the mitochondrial morphology in HUVECs. Cells were subjected to super-resolution confocal microscopy. Scale bar = 10 μm. Data are shown as the mean ± S.D. of three independent experiments.

Iron overload is usually accompanied by mitochondrial oxidative stress, leading to mitochondrial dysfunction^26^. The typical cell morphology of ferroptosis is smaller mitochondria with condensed mitochondrial membrane densities, reduction or vanishing of mitochondria crista, as well as outer mitochondrial membrane rupture^6^. Currently, ZnO NPs-treated cells showed shrunk mitochondria and fused mitochondrial cristae (Fig. 3a), which are reminiscent of ferroptotic cancer cells observed in response to erastin^4,27,28^. One of the direct molecular targets of erastin is the mitochondrial voltage-dependent anion channel (VDAC) protein, which mediates mitochondrial iron uptake and enhances ferroptosis^27^. Indeed, VDAC2 and VDAC3 expression was considerably upregulated with ZnO NPs (Fig. 3b, c). The ZnO NPs-treated group showed a large number of fragmented, smaller and short-tubular mitochondria that resemble erastin treatment (Fig. 3d).

Mitochondria are highly dynamic organelles with variable morphology, number and distribution within cells. Mitochondrial fusion includes the fusion of the outer and inner membranes, which is manipulated by three dynamin-related GTPases, i.e., mitofusin 1 (MFN1), MFN2, and optic atrophy 1 protein (OPA1). Mitochondrial fission is mainly controlled by dynamin-related proteins (DRPs) in eukaryotes, members of which are large self-assembling GTPases. The downregulation of the MFN1 and OPA1 genes, as well as the upregulation of the DRP1 gene (Supplementary Fig. 15) by ZnO NPs, explains the fragmented mitochondria. This result is consistent with the conclusions from TEM and MitoTracker® Deep Red FM staining. Together, these results further demonstrated that ZnO NPs-induced ferroptosis is a mitochondrial-driven cell death.

### P53 functions as a pivotal master gene in ZnO NPs-induced ferroptosis

**Fig. 4:**
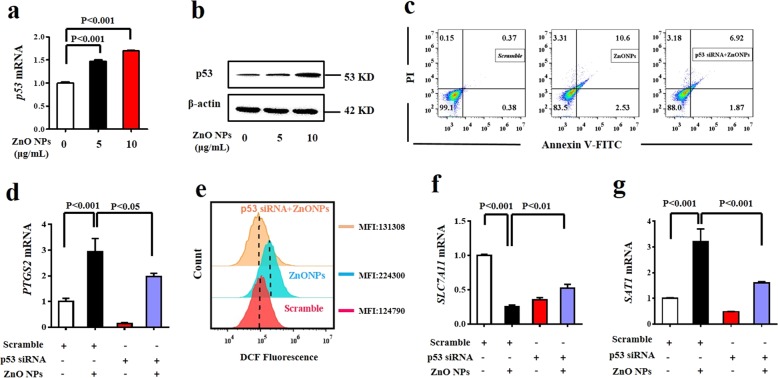
P53 functions as a pivotal master gene in ZnO NPs-induced ferroptosis. HUVECs were treated with ZnO NPs (5 or 10 μg/mL) for 24 h. **a** Total RNA was extracted for p53 mRNA analysis using qRT-PCR. **b** Western blotting analysis for the expression of p53. HUVECs were transiently transfected with p53 siRNA and subjected to the following analysis. **c** Annexin-V/PI staining. **d** PTGS2 mRNA analysis. **e** ROS determination by DCFH-DA probe, MFI: mean fluorescence intensity. **f** SLC7A11 mRNA analysis and **g** SAT1 mRNA analysis. Data are shown as the mean ± S.D. of three independent experiments.

A pioneering study by Jiang et al. implicated that p53 sensitizes cells to ferroptosis^29^. P53 acetylation is crucial for its activity mediating ferroptosis^30^. Interestingly, the activation of p53 by ZnO NPs has been reported in different occasions^31–34^. qRT-PCR and western blotting results showed that ZnO NPs increased p53 mRNA and protein expression, respectively (Fig. 4a, b). A reduced apoptosis rate was achieved using p53 siRNA in ZnO NPs-treated HUVECs, further illustrating that the abrogation of p53 enhanced cellular tolerance toward ZnO NPs (Fig. 4c). The induction of p53 was presumed to be associated with oxidative stress^34^. After inhibiting p53 expression using siRNA, we found that the mRNA level of the ferroptosis biomarker PTGS2 was significantly downregulated by ZnO NPs (Fig. 4d). DCFH-DA analysis showed that p53 siRNA reduced intracellular ROS levels (Fig. 4e).

GSH depletion and lipid peroxidation are major hallmarks of ferroptotic cell death. P53 binds to the system X_c_^−^ transporter subunit cystine/glutamate transporter (SLC7A11) and negatively regulates SLC7A11 by decreasing cystine import and reducing GSH levels^29,35,36^. ZnO NPs-treated group had decreased SLC7A11 mRNA level compared with the control group (Fig. 4f). Again, ZnO NPs exposure increased the level of SAT1 while p53 siRNA abrogated this effect (Fig. 4g). The protein expression levels of SLC7A11 and SAT1 correspond to their mRNA levels (Supplementary Fig. 16). SAT1 siRNA treatment inhibited ALOX15 and PTGS2 mRNA levels, suggesting the inhibition of ferroptosis, whereas no increase was observed in the levels of the other two lipoxygenases, arachidonate 5-lipoxygenase (ALOX5) and arachidonate 12-lipoxygenase (ALOX12) (Supplementary Fig. 17). Notably, although accumulating evidence supports the activity of p53 in the regulation of ferroptosis, p53 alone is incapable of inducing ferroptosis^37^.

### Particle remnants and Zn^2+^ ions both contribute to ZnO NPs-induced ferroptosis

In addition to reported variations in cytotoxicity, there is no consensus on the underlying mechanisms that drive the toxicity of ZnO NPs, i.e., the ZnO particles per se, the released Zn^2+^ or their combination. Zn^2+^ is released from the surface of ZnO NPs when they are suspended in aqueous state, both in medium and organelles with low pH^38^.

**Fig. 5:**
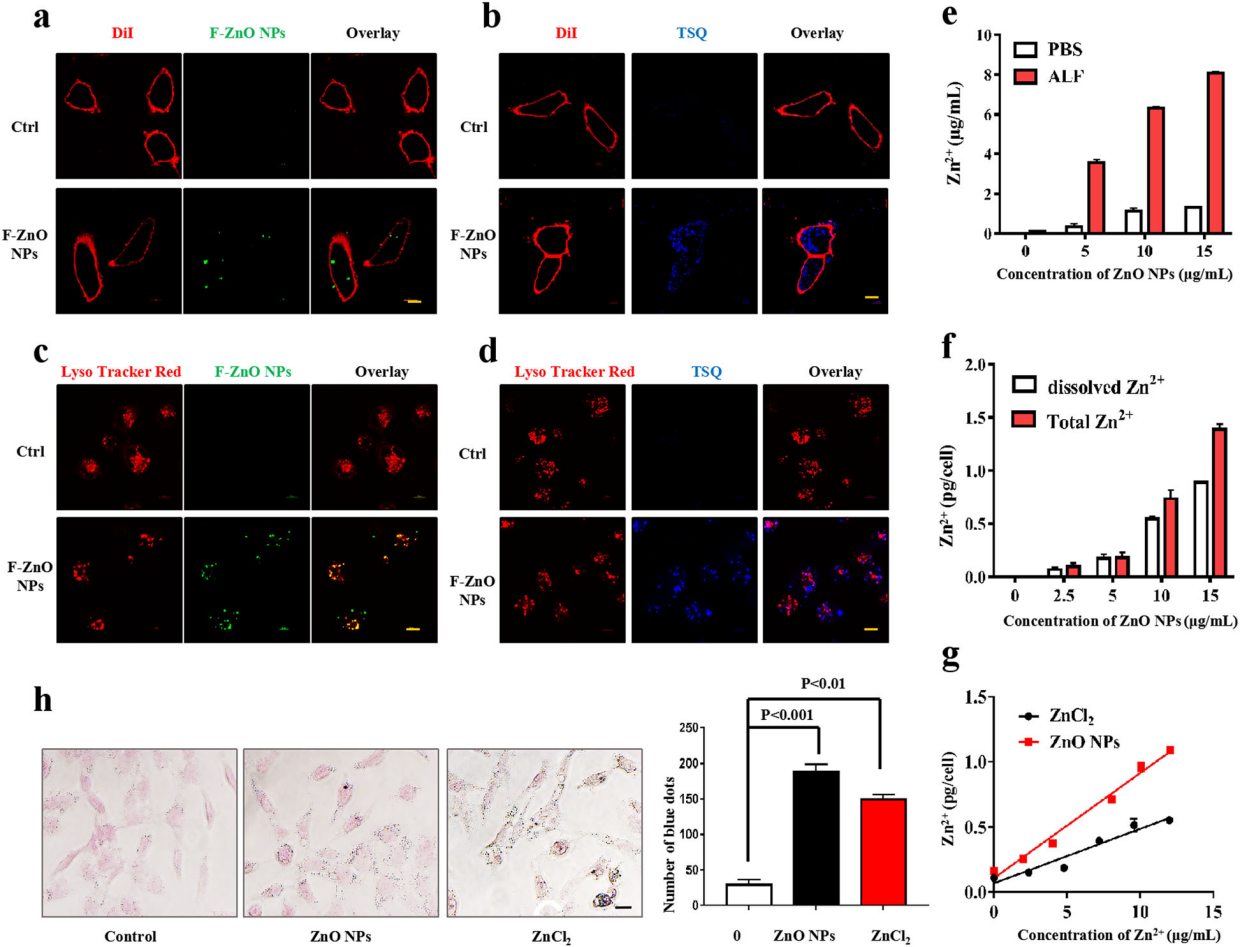
Particle remnants and Zn^2+^ ions both contribute to ZnO NPs-induced ferroptosis. Super-resolution confocal microscopy was used to study the uptake of F-ZnO NPs and the cellular distribution of Zn^2+^. HUVECs were treated with F-ZnO NPs at concentrations of 10 μg/mL for 24 h. After permeabilization and fixation, cells were stained with **a** the cellular membrane marker DiI (20 μM), **b** DiI (20 μM) and Zn^2+^-specific fluorescent dye TSQ (30 μM), **c** Lyso Tracker Red (1 μM), or **d** Lyso Tracker Red (1 μM) and TSQ (30 μM). Then, cells were subjected to super-resolution confocal microscopy. Scale bar = 10 μm. **e** ZnO NPs were exposed to PBS at pH 7.4 and artificial lysosomal fluid (ALF) at pH 4.5 for 24 h and the free Zn^2+^ content was analyzed by AAS. **f** HUVECs were exposed to ZnO NPs for 24 h, and the dissolved Zn^2+^ and total Zn^2+^ contents were analyzed by AAS. Zn^2+^ content is expressed as pg/cell. **g** HUVECs were exposed to ZnO NPs or ZnCl_2_ for 24 h and the dissolved Zn^2+^ content was analyzed by AAS. Zn^2+^ content is expressed as pg/cell. The results are presented as the mean ± S.D. of four replicates. **h** HUVECs were exposed to 10 μg/mL ZnO NPs or 31.9 μg/mL ZnCl_2_ (stoichiometric equivalent of Zn^2+^ inside the cells, compared with 10 μg/mL ZnO NPs) for 24 h. Lillie divalent iron staining analysis was performed. Scale bar = 20 μm.

To more precisely follow particle processing behavior in cells, fluorescent-labeled F-ZnO NPs were generated by grafting the particle surface with (3-aminopropyl)triethoxysilane (APTS), followed by the addition of the amine-reactive dye fluorescein isothiocyanate (FITC)^39^. Green fluorescent spots indicated the uptake of F-ZnO NPs by cells (Fig. 5a). To test whether ZnO NPs-induced ferroptosis is associated with Zn^2+^ release inside cells, we performed cellular staining with Zn^2+^-specific fluorescent dye N-(6-methoxy-8-quinolyl)-*p*-toluenesul fonamide (TSQ)^40^. Compared with the low intensity staining of untreated HUVECs, F-ZnO NPs-treated cells showed a generalized increase in blue fluorescence with prominent staining of the cellular membrane that was stained by DiI, suggesting that Zn^2+^ derived from the particle remnants concentrates in the cells (Fig. 5b). Next, we discuss the subcellular localization of F-ZnO NPs in cells^41^. After incubation with F-ZnO NPs for 24 h, F-ZnO NPs were colocalized with lysosomal fluorescent spots, resulted in a composite orange fluorescence profile (Fig. 5c). In addition, the overlap of fluorescent signals from TSQ and Lyso Tracker Red suggested that Zn^2+^ derived from the particles concentrates in the lysosomal compartment (Fig. 5d).

We then attempted to mimic the intracellular behavior of ZnO NPs by investigating Zn^2+^ release in PBS at pH 7.4 and artificial lysosomal fluid (ALF) at pH 4.5. As presented in Fig. 5e, the overall amount of released Zn^2+^ present in PBS solution was considerably lower than corresponding amount measured in ALF. At the highest concentration of ZnO NPs exposure (15 µg/mL), Zn^2+^ concentrations corresponding to the dissolution of the NPs were 54% (in ALF) and 11% (in PBS). This result implied the massive degradation of ZnO NPs in lysosomes. For comparative purposes, the concentration of Zn^2+^ inside the cell was also evaluated. The measurement resulted in an average Zn^2+^ concentration per cell after 24 h of ZnO NPs incubation. The supernatant zinc concentration represented dissolved Zn^2+^, while the total zinc (including particle remnants and dissolved Zn^2+^) was achieved by acidification (Fig. 5f). From the data of the dissolved Zn^2+^ to total Zn^2+^ ratio, we conclude that the majority (~60%) of ZnO NPs rapidly degraded into Zn^2+^ after entering the cells (presumably in the acidic lysosomal compartment), which is similar to that found in the ALF simulation. The dissolution of ZnO NPs into Zn^2+^ is dependent on the properties of the particles as well as the media^42^.

Zinc is mostly bound to proteins or sequestered in lysosomes; however, no Zn^2+^ signal in the control group indicates that intrinsic zinc does not affect this measurement (Fig. 5f). To rule out a surface effect of ZnO NPs, i.e., the formation of ZnO NPs agglomerates and interaction with the cell membrane, we compared the uptake of particle (ZnO NPs) and dissolved Zn^2+^ (ZnCl_2_), at concentrations normalized to Zn^2+^ ion. From Fig. 5g, ZnO NPs show nearly 2-fold efficiency for internalization of Zn^2+^ compared with ZnCl_2_. The current data clearly demonstrate that HUVECs take up both particles and dissolved Zn^2+^. Divalent ion chelators, e.g., DFO, are not suitable for the elimination of free Zn^2+^ because they chelate Fe^2+^ spontaneously. To determine whether Zn^2+^ induces ferroptosis similar to ZnO NPs, HUVECs were treated with ZnCl_2_, calibrated by the amount of Zn^2+^ in the cells. Figure 5h shows that Zn^2+^ induces comparable staining when compared with stoichiometric equivalent ZnO NPs (inside the cells), which confirms that Zn^2+^ induces ferroptosis by direct ZnCl_2_ exposure. To further nail the effect of dissolved Zn^2+^ on ferroptosis, a set of parallel experiments were conducted by direct exposure of cells to ZnCl_2_ (equal amount of Zn^2+^ in the cells) and comparable results were obtained, compared with ZnO NPs (Supplementary Fig. 18).

The amount of Zn^2+^ leached from the ZnO NPs then taken up by cells seems to be insignificant, as the majority of dissolved Zn^2+^ was found in the acidic compartment (Fig. 5e) and the internalization of zinc was weakened for Zn^2+^ form (Fig. 5g). Therefore, in the case of ZnO NPs exposure, dissolved Zn^2+^ plays the dominant role in ferroptosis. The dynamic equilibrium of zinc dissolution is not discussed here, therefore, an independent study demonstrated that the dissolution of ZnO NPs reached 80% of the maximum dissolved Zn^2+^ within 3 h^43^. Together, it is safely concluded that the ZnO NPs-induced ferroptosis is primarily due to the enhancement of the intracellular concentration of Zn^2+^.

### Ferroptosis is a general form of cell death induced by NMs

**Fig. 6:**
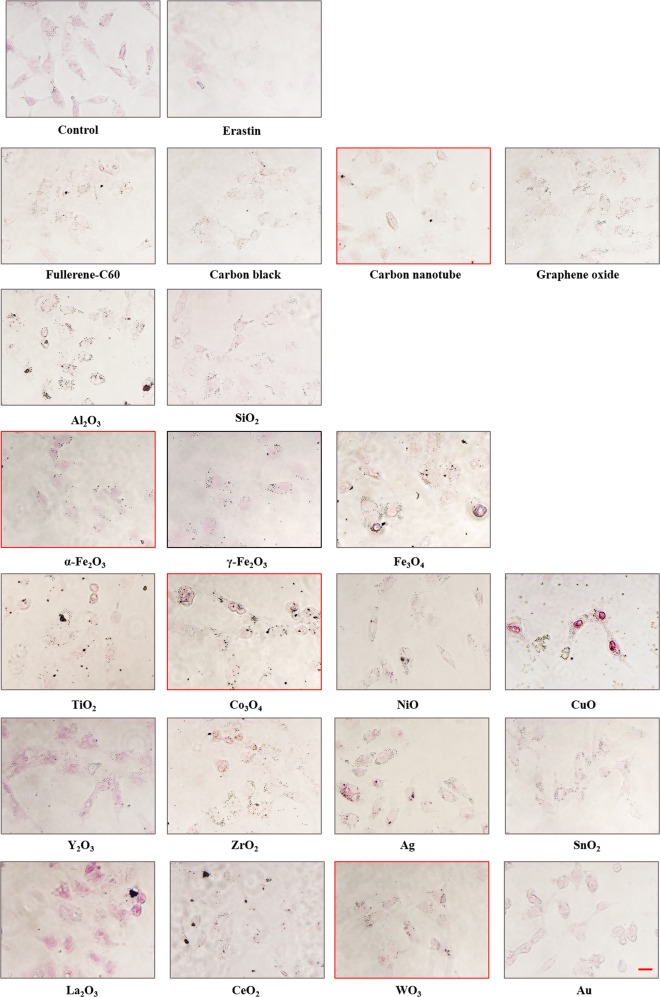
Nanomaterials cause intracellular Fe^2+^ elevation in HUVECs. HUVECs were exposed to 10 μg/ml of listed nanomaterials or 25 μM erastin for 24 h, and subjected to Lillie divalent iron staining. Red circled images indicated insignificant staining. Scale bar = 20 μm.

We next ask whether only ZnO NPs, or only ion-leaking NPs, has the ability of triggering ferroptosis. Therefore, we performed an intracellular ferrous iron staining analysis with 21 different NMs. Most of the NMs used are 0-dimensional, but there are also 1-dimensional carbon nanotubes and 2-dimensional graphene oxide (Supplementary Table 2). The primary size of these NMs are between 5 and 200 nm. These NMs are positively or negatively charged with good dispersities, both in PBS and in RPMI1640 medium. Most of NMs have a certain degree of influence on the accumulation of iron in HUVECs, suggesting a specific proferroptotic effect of NMs (Fig. 6), and related quantification was provided in Supplementary Fig. 19. This effect cannot be attributed to metal ions released from the NPs. Although a similar downstream cell-death phenotype was found, ferroptosis inducers may activate different signal pathway; for example, erastin modulates VDAC2/3^27^ and system X_c_^− ^^4^ to trigger ferroptosis, while Ras-selective lethal small molecule 3 (RSL 3) does not affect these factors^44^. As shown, most of NMs upregulated ferroptotic gene expressions (Supplementary Fig. 20), however, whether these NMs with different characteristics (composition, size, shape, and surface charge) share the same ferroptotic mechanism as ZnO NPs is currently unknown. To prove the universal principle that applies in ZnO NPs-induced cell death, multiple cell lines with different resources were tested. The results resemble those found with HUVECs in appearance (Supplementary Fig. 21). Nevertheless, the nature of the death signal responsible due to ZnO NPs exposure (along with other NMs) has not been completely defined. Further investigation would be beneficial for improving the understanding of the mechanisms governing NMs-induced cell death.

## Discussion

As usage of NMs rapidly grows, it is urgent and important to assess their safety. Previous in vitro and in vivo studies demonstrated the association of ZnO NPs exposure with various cell-death pathways; however, whether ZnO NPs, along with other “iron free” NMs induce ferroptosis has not been reported. In the present work, we identified that ferroptosis is a general cell death caused by NMs. The occurrence of ferroptosis is evidenced biologically, genetically, and morphologically.

The most important finding in the current study is, unlike previous findings, our data support that “iron free” NMs trigger ferroptosis. Thus, we attempt to address its fundamental principle. Considering the different compositions and properties (size, shape, and zeta potential) of NMs, the common mechanism that leads to ferroptosis is excess ROS. After their internalization, NMs favorably mobilize to mitochondria and dysregulate the mitochondrial antioxidant defense system. Alternatively, the proinflammatory effect is an intrinsic nature of NMs that may cause ROS. These two mechanisms are applied to the majority of NMs regardless of their composition or other characteristics. Although gold and silver-constituent NMs are relatively chemically inert, their exposures to cells are also linked with the promotion of ROS. Another vital finding in the current study is that ZnO NPs-induced dysregulation of iron homeostasis. Elements have redox properties, such as iron, may catalyze the production of ROS. By taking advantage of the Fenton reaction and Haber-Weiss reaction, iron-oxide-based NMs were designed and synthesized for anticancer therapy^45,46^. In fact, numerous examples of damage to cells, in which iron are implicated. However, “iron free” ZnO NPs do not provide iron essentially, the source of iron under physiological condition, where iron availability is relative low, need to be discussed. Lysosomes (the main NMs-targeting organelle) and mitochondria may release iron from the LIP. Although ZnO NPs exhibited rather complicated regulation on iron uptake, storage and export related gene expressions, however, an overall consequence of ZnO NPs exposure is iron accumulation, which implied that the iron-dysregulation mechanism plays an important role in ZnO NPs-induced ROS formation. Alternatively, ROS-mediated mitochondrial damage may defect heme and iron–sulfur cluster-containing proteins synthesis, and in turn accumulates “free” iron in mitochondria. Therefore, ROS formation and the dysregulation of iron homeostasis is an interdependent event. Dixon and Stockwell summarized how iron and ROS contribute to a variety of cell-death pathways, including ferroptosis^5^. Of note, we have no intention to attribute NMs-induced ferroptosis solely to ROS, individual investigations of different NMs are encouraged.

The third interesting finding in the current study is that ZnO NPs-induced dysregulation of iron homeostasis partially due to dissolved Zn^2+^. Although Zn^2+^ is an inert cation and does not undergo a redox reaction, Zn^2+^ overload is closely related to ROS and subsequently mitochondrial injuries^47,48^. The aberrant homeostasis of transition metal ions, e.g., Fe^2+^/Fe^3+^, Zn^2+^, and Ca^2+^, plays important roles in the pathogenesis of various diseases. For instance, a recent study has highlighted the interaction of Zn^2+^ homeostasis and ROS signaling suggesting their interdependence^49^. Mitochondria contain dynamic pools of these metal ions that are incorporated into corresponding metalloproteins^50^. Thus, Zn^2+^ overload-triggered mitochondrial injuries may exaggerate the dysregulation of dynamic metal pools (including the LIP), resulting in the elevation of intracellular iron and ultimately ferroptosis. Interestingly, both ZnO NPs and Zn^2+^ were reported to disrupt intracellular Ca^2+^ homeostasis, which activates Ca^2+^-dependent pro-death signaling^51–53^. Our current results facilitate the understanding of the cell-death mechanism involved in the disruption of metal ion homeostasis.

A recent review summarized the effect of p53 on the regulation of ferroptosis network^54^. Specifically, the pro-death function of p53 in ferroptosis was mutually found in previous^29^ and current study, includes the inhibition of SLC7A11 expression and the promotion of SAT1 expression. On the contrary, the pro-survival function of p53 in ferroptosis have been also reported^54^, suggested the bipolar regulation of p53 on cell death. Interestingly, there are several reports in which zinc can either block or accelerate apoptosis, due to different experimental settings. Under mild ROS conditions, p53 stimulates the expression of antioxidant genes to restore oxidative homeostasis, whilst extreme ROS level initiate apoptosis through p53^34^. Notably, the activation of p53 may in turn induce ROS production by regulating certain genes^55^. ZnO NPs-induced signaling that associated with p53 has been reviewed^11^. At last but not least, the current study, for the first time, demonstrated that ZnO NPs-induced ferroptosis is p53-dependent; however, whether p53 has other functions in ZnO-induced ferroptosis is unknown. For instance, other work reported that acute exposure of eukaryotes to Zn^2+^ decreased intracellular iron content; however, no information regarding to p53 regulation and ferroptosis has been released^56^. In addition, it is commonly accepted that p53 involved in multiple line of cell death, including apoptosis, necrosis and autophagy, however, the activities of p53 in ZnO NPs (or Zn^2+^)-induced cell death are indistinct; since several cell death share ROS/p53 axis collectively, whether these are pivotal molecules that manipulate the cross talk of these cell death is not currently understood.

**Fig. 7:**
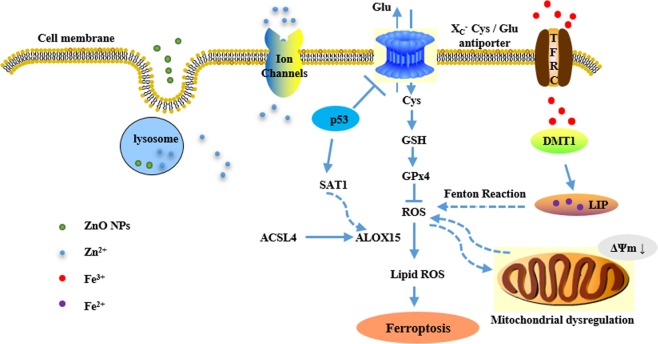
Proposed mechanism of ZnO NPs-induced ferroptosis. Ferroptosis is initiated via various signal pathways, such as Fe^2+^ accumulation, glutathione depletion and lipid peroxidation. The ZnO NPs can be endocytosed into lysosomal compartments where ZnO dissolves and releases zinc ions into the cytoplasm. ZnO NPs and zinc ions in the cytoplasm can activate p53, which may repress the transcription of SLC7A11, a component of the cystine/glutamate antiporter and glutathione depletion, and trigger SAT1 gene expression is enhanced in the presence of activated p53. Similarly, ZnO NPs and zinc ions in the cytoplasm can also impair organelles such as mitochondria and disrupting iron metabolism. and subsequent ferroptosis can occur by over-accumulation of lipid ROS.

In summary, the current study demonstrated that ZnO NPs-induced cell death coincides with the definition of ferroptosis. We conclude that HUVECs death induced by ZnO NPs includes ferroptosis, in addition to apoptosis, autophagy, and necroptosis. ZnO NPs cause ferroptosis by disrupting iron metabolism (iron overload), mitochondrial dynamics (increased mitochondrial fission) and redox homeostasis (GSH depletion and lipid peroxidation generation), which appears to be a p53-driven process (mechanism summarized in Fig. 7). This is the first comprehensive investigation on the role of “iron free” NMs in ferroptotic cell death, which emphasizes the importance of understanding NMs-induced cell death.

## Supplementary information

Supplementary Information
