# Supplementary material for: “Iron free” zinc oxide nanoparticles with ion-leaking properties disrupt intracellular ROS and iron homeostasis to induce ferroptosis
Source: Cell Death Dis. 2020 Mar 13;11(3):183. doi: 10.1038/s41419-020-2384-5 (PMC7070056; doi:10.1038/s41419-020-2384-5)
Supplement: Supplementary file 1 — Supplementary Information [file 41419_2020_2384_MOESM1_ESM.docx]

**Supplementary for**

**“Iron Free” Zinc Oxide Nanoparticles with Ion-Leaking Properties Disrupt Intracellular ROS and Iron Homeostasis to Induce Ferroptosis**

Changping Zhang^1^, Zixuan Liu^2^, Yuhao Zhang^1^, Liang Ma^1^, Erqun Song^2^, Yang Song^2,*^

^1^Key Laboratory of Luminescence and Real-Time Analytical Chemistry (Southwest University), Ministry of Education, College of Food Science, Southwest University, Chongqing, People’s Republic of China, 400715

^2^Key Laboratory of Luminescence and Real-Time Analytical Chemistry (Southwest University), Ministry of Education, College of Pharmaceutical Sciences, Southwest University, Chongqing, People’s Republic of China, 400715

^*^To whom correspondence should be addressed

**Y. S.:** Key Laboratory of Luminescence and Real-Time Analytical Chemistry (Southwest University), Ministry of Education, College of Pharmaceutical Sciences, Southwest University, Chongqing, People’s Republic of China, 400715. Tel: +86-23-68250371. Fax: +86-23-68251225. E-mail addresses: ysong@swu.edu.cn or songyangwenrong@hotmail.com

###

### Supplementary Fig. 1.

TEM were performed to observe the NPs morphology and sizes. NPs were sonicated for 10 minutes in water and drop cast on a TEM grid prior to imaging. (a) ZnO NPs and (b) F-ZnO NPs.

**
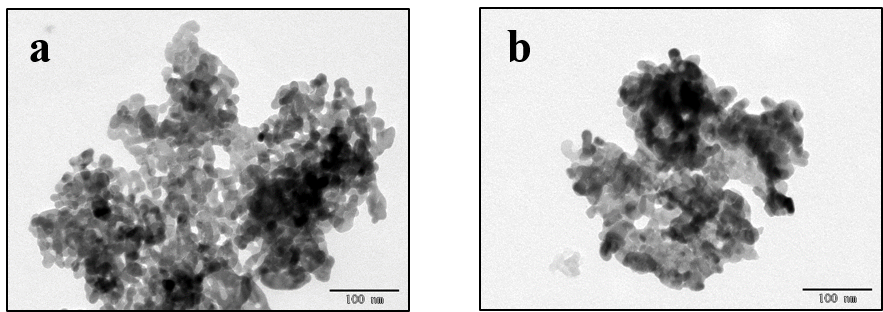
**

### Supplementary Fig. 2.

ZnO NPs was characterized by X-ray diffraction (XRD) with CuKα radiation, revealed a crystalline nature structure of ZnO NPs which is consistent with the standard Zincite, JCPDS 5-0664.

**
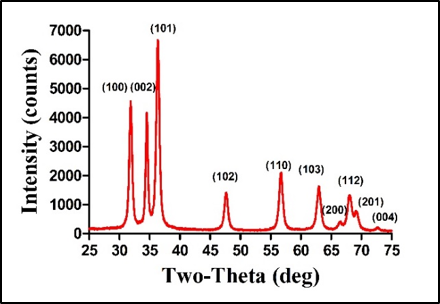
**

### Supplementary Fig. 3.

Cell viability analysis of HUVECS after incubation with ZnO NPs (5, 10 or 15 μg/mL) for 24 h. Data are shown as the mean ± S.D. of three independent experiments.


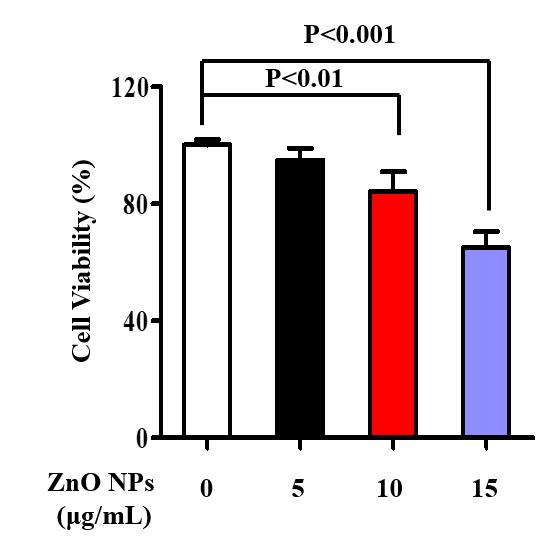


### Supplementary Fig. 4.

HUVECs were treated with ZnO NPs (5, 10 or 15 μg/mL) for 24 h. Cell death was calculated from lactate dehydrogenase (LDH) leakage. Data are shown as the mean ± S.D. of three independent experiments.

**
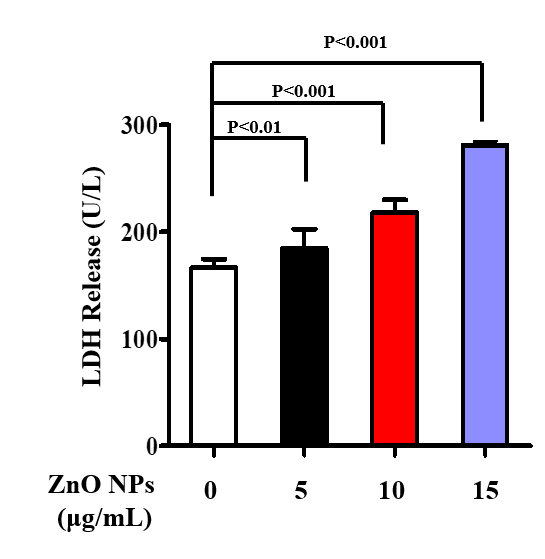
**

### Supplementary Fig. 5.

HUVECs were treated with ZnO NPs (5, 10 or 15 μg/mL) for 24 h and stained by adding Annexin V/PI staining to the culture medium.

**
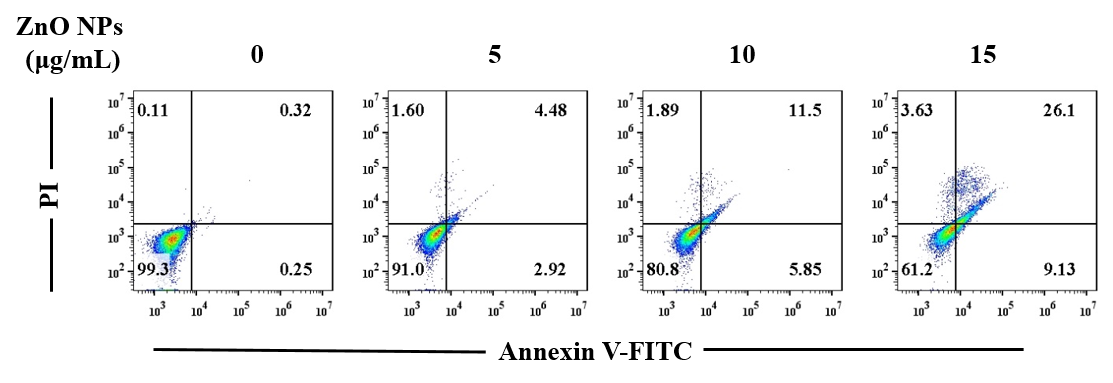
**

### Supplementary Fig. 6.

HUVECs were treated with ZnO NPs (5, 10 or 15 μg/mL) for 24 h and stained by AO-EB staining to the culture medium. Scale bar = 200 μm.

**
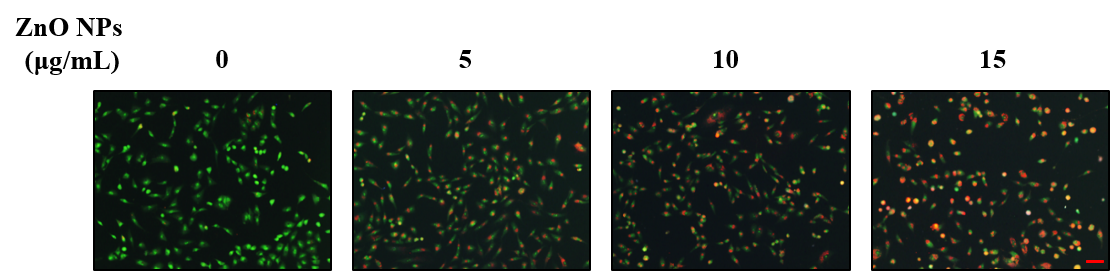
**

### Supplementary Fig. 7.

HUVECs were treated with ZnO NPs (5, 10 or 15 μg/mL) or CCCP (50 μM) for 24 h. Loss of mitochondrial membrane potential (ΔΨm) after staining with JC-1 probe. Cells were analyzed through flow cytometry. Data were shown as the mean ± S.D. of three independent experiments.

**
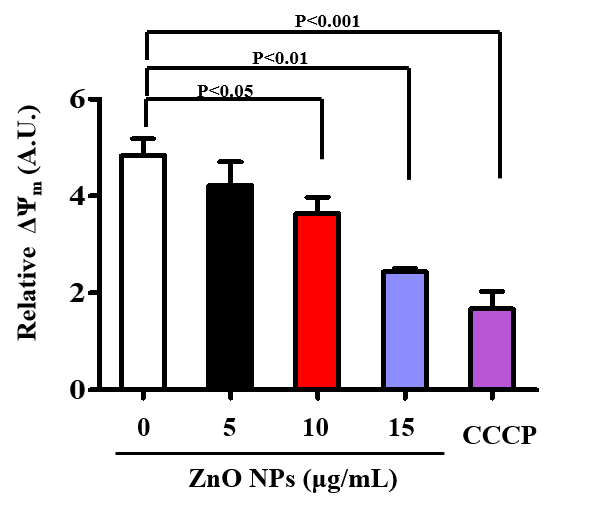
**

### Supplementary Fig. 8.

HUVECs were treated with ZnO NPs (5 or 10 μg/mL) for 24 h. Western blotting analysis for the expressions of cleaved caspase 8 and caspase 3.

**
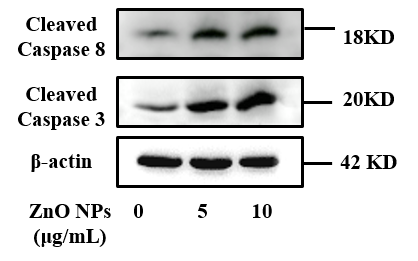
**

### Supplementary Fig. 9.

HUVECs after exposure to ZnO NPs (10 μg/mL) for 24 h. The addition of specific cell-death inhibitors [including ferroptosis inhibitors Ferrostatin-1 (Fer-1, 10 μm), Deferoxamine (DFO, 100 μm) and Liproxstatin-1 (LIP-1, 50 nM); necroptosis inhibitors Necrostatin 1 (Nec-1, 10 μM) and (E)-Necrosulfonamide (NSA, 1 μM); caspase inhibitor Z-VAD-FMK (20 μM); autophagy inhibitors 3-methyladenine (3-MA, 2 mM) and chloroquine (CQ, 10 μM)] decreased the relative PI fluorescence in the presence of 10 μg/mL ZnO NPs. Data were shown as the mean ± S.D. of three independent experiments.

**
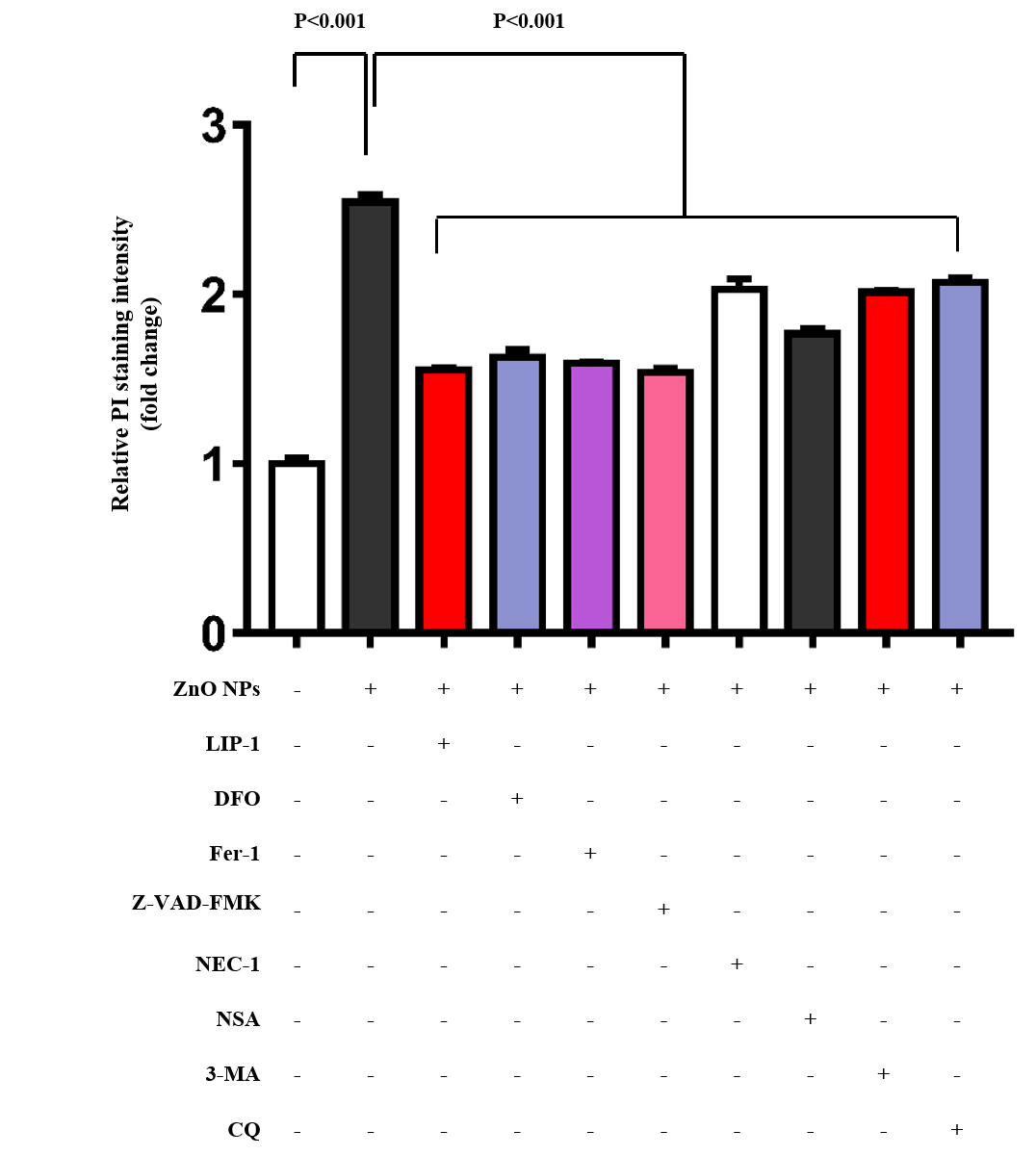
**

### Supplementary Fig. 10.

ZnO NPs decreased GPx4 protein level. HUVECs were treated with ZnO NPs (5 or 10 μg/mL) for 24 h and total protein lysates were subjected to Western blotting analysis.


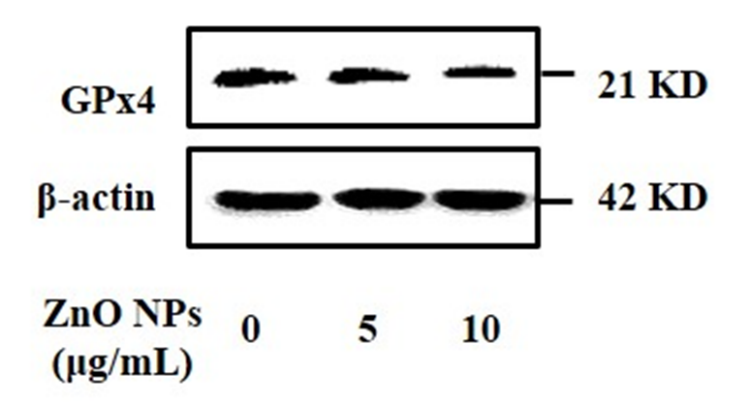


### Supplementary Fig. 11.

HUVECs transiently transfected with corresponding siRNA for 6 h in the presence of 10 μg/mL ZnO NPs, the generation of ROS was determined by the DCFH-DA probe. (a) ACSL4 and (b) ALOX15. MFI: Mean Fluorescence Intensity

**
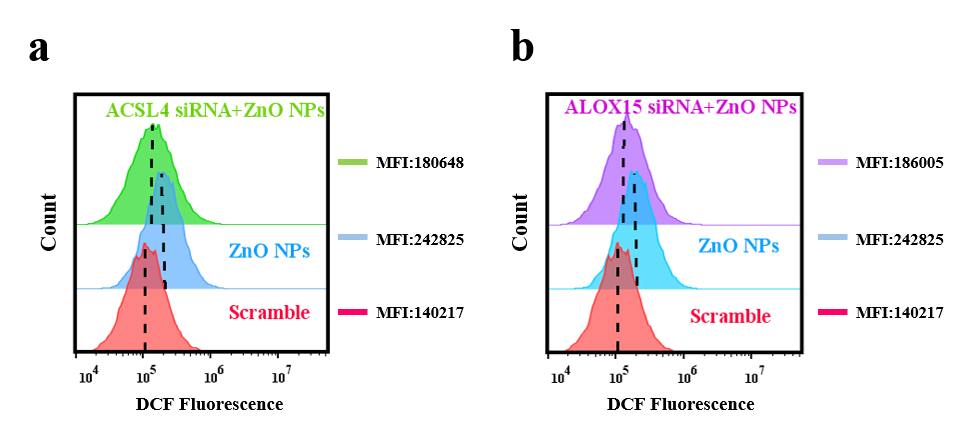
**

### Supplementary Fig. 12.

Evidence of ferroptosis in HUVECs after treatment with erastin (25 μM) for 24 h. (a) Cell viability analysis. (b) LDH leakage analysis. Data were shown as the mean ± S.D. of three independent experiments. (c) Western blotting analysis for the expression of FTL, FTH, TFRC, GPx4 and β-actin.

**
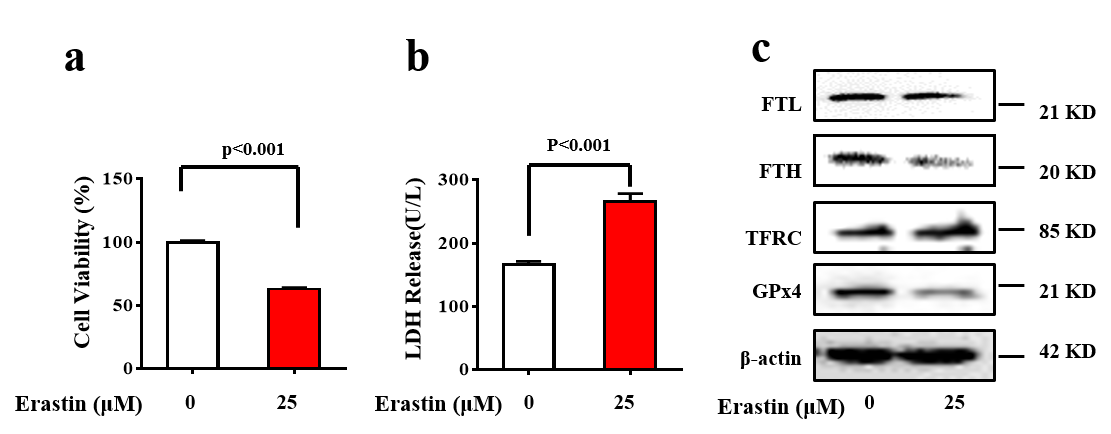
**

### Supplementary Fig. 13.

HUVECs were treated with ZnO NPs (5 or 10 μg/mL) for 24 h and total RNA was extracted for the analysis of (a) MZF1 and (b) BACH1 mRNA levels. Data were shown as the mean ± S.D. of three independent experiments.

**
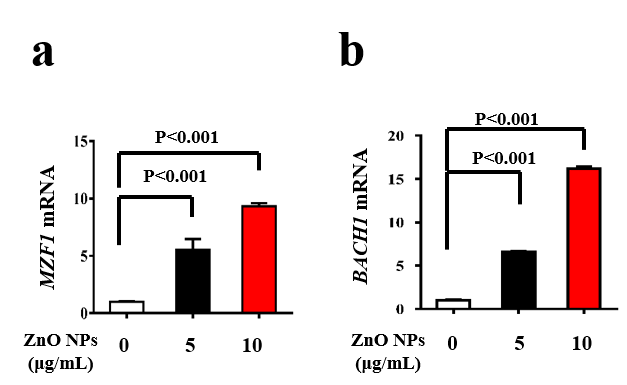
**

### Supplementary Fig. 14.

HUVECs were treated with ZnO NPs (5 or 10 μg/mL) for 24 h and total protein lysates were subjected to Western blotting analysis.

**
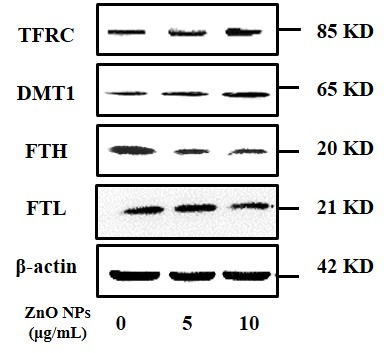
**

### Supplementary Fig. 15.

HUVECs were treated with ZnO NPs (5 or 10 μg/mL) for 24 h and total RNA was extracted for the analysis of (a) MFN1, (b) OPA1 and (c) DRP1 mRNA levels. Data were shown as the mean ± S.D. of three independent experiments.


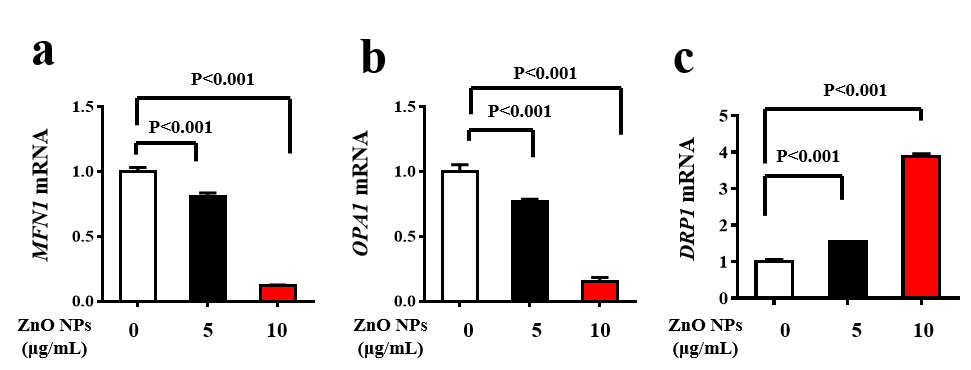


### Supplementary Fig. 16.

HUVECs were treated with ZnO NPs (5 or 10 μg/mL) for 24 h and total RNA was extracted for mRNA analysis using qRT-PCR. (a) SLC7A11 mRNA. (b) SAT1 mRNA. Data are shown as the mean ± S.D. of three independent experiments). (c) Western blotting analysis for the expressions of SLC7A11 and SAT1.

**
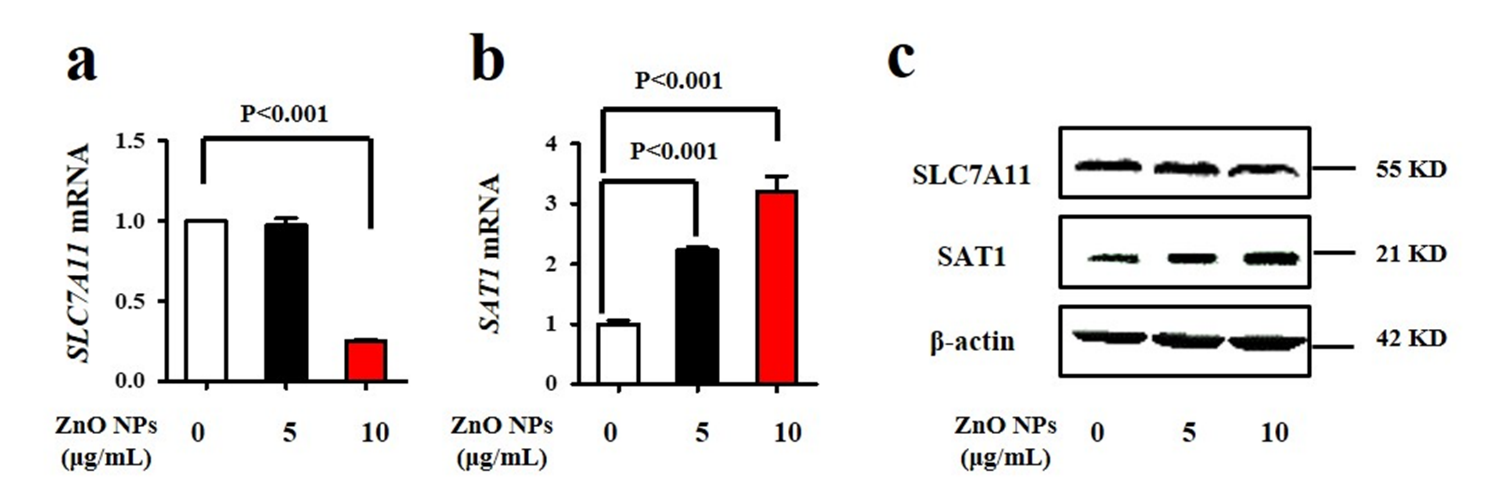
**

### Supplementary Fig. 17.

HUVECs were treated with ZnO NPs (5 or 10 μg/mL) for 24 h with or without SAT1 siRNA treatment. Total RNA was extracted for mRNA analysis using qRT-PCR. (a) ALOX15 mRNA. (b) PTGS2 mRNA. (c) ALOX5 mRNA and (d) ALOX12 mRNA. Data are shown as the mean ± S.D. of three independent experiments.

**
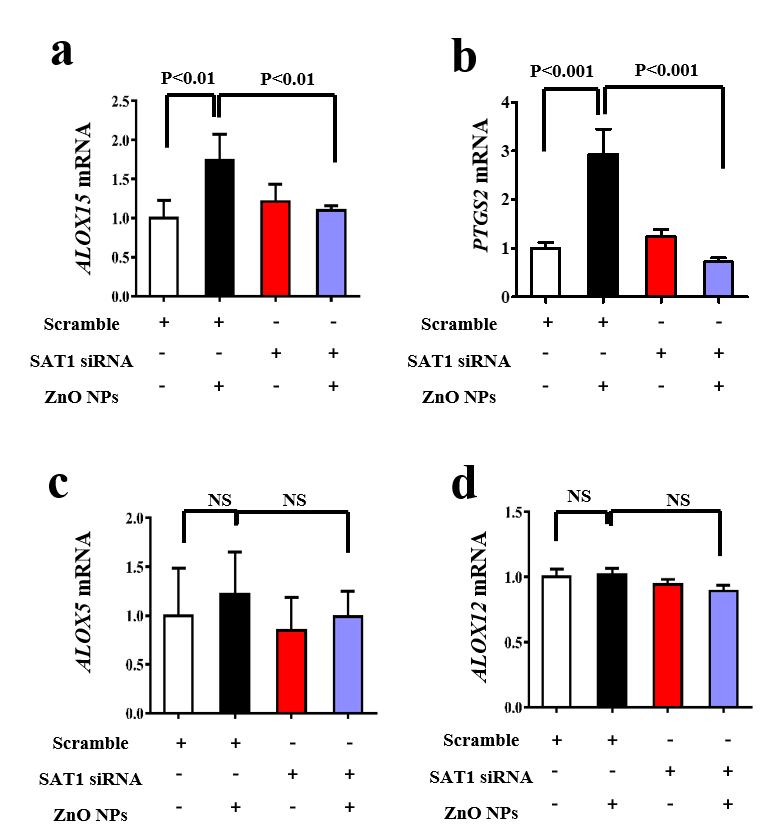
**

### Supplementary Fig. 18.

ZnCl_2_ triggers ferroptosis. HUVECs were treated with ZnCl_2_ (31.7 μg/mL, which has equivalent intracellular Zn^2+^ compare with 10 μg/mL of ZnO NPs) for 24 h. (a) GSH, (b) GPx, (c) ROS and (d) MDA levels were measured. Total RNA was extracted for the analysis of mRNA levels of interest using qRT-PCR. (e) ALOX15, (f) ACSL4, (g) GPx4, (h) PTGS2, (i) p53, (j) SLC7A11 and (k) SAT mRNA. Data are shown as the mean ± S.D. from three independent experiments. (l) Western blotting analysis for the expression of SAT1, p53, GPx4, SLC7A11 and FTH proteins.


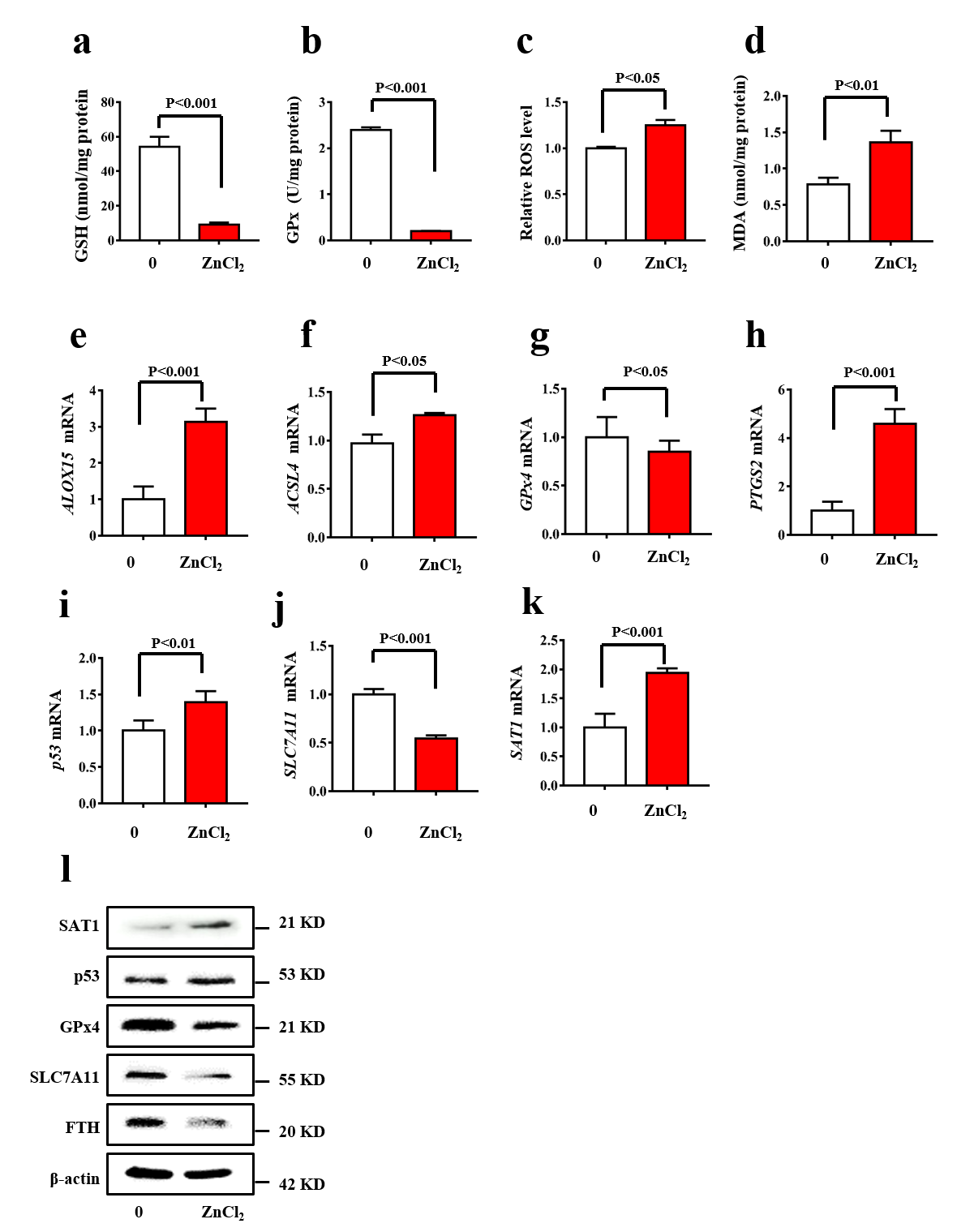


### Supplementary Fig. 19.

ZnO NPs cause intracellular Fe^2+^ elevation in various cell lines. Quantification of blue dots were shown.


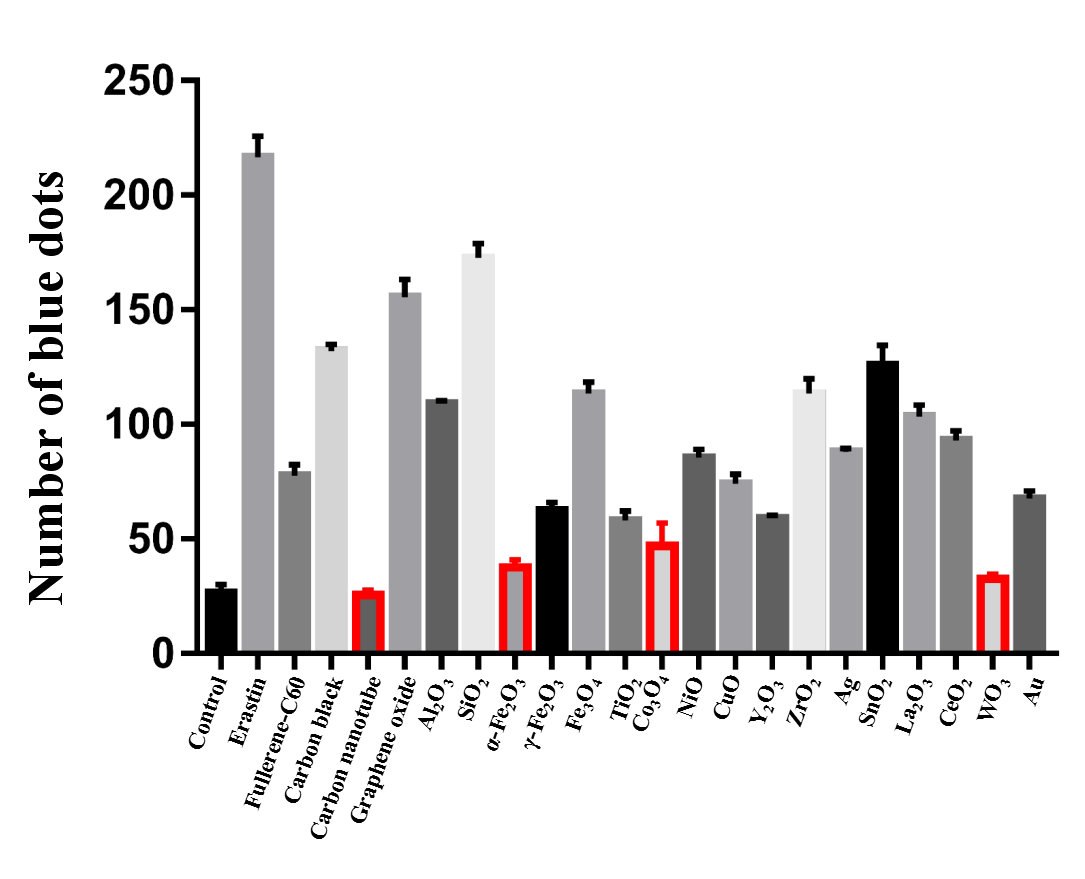


### Supplementary Fig. 20.

HUVECs were treated with various NMs (10 μg/mL) for 24 h and total RNA was extracted for mRNA analysis using qRT-PCR. (a) PTGS2. (b) GPx4 and (c) ACSL4. Data are shown as the mean ± S.D. of three independent experiments. *P<0.05, **P<0.01, ***P<0.001, ns=no significance


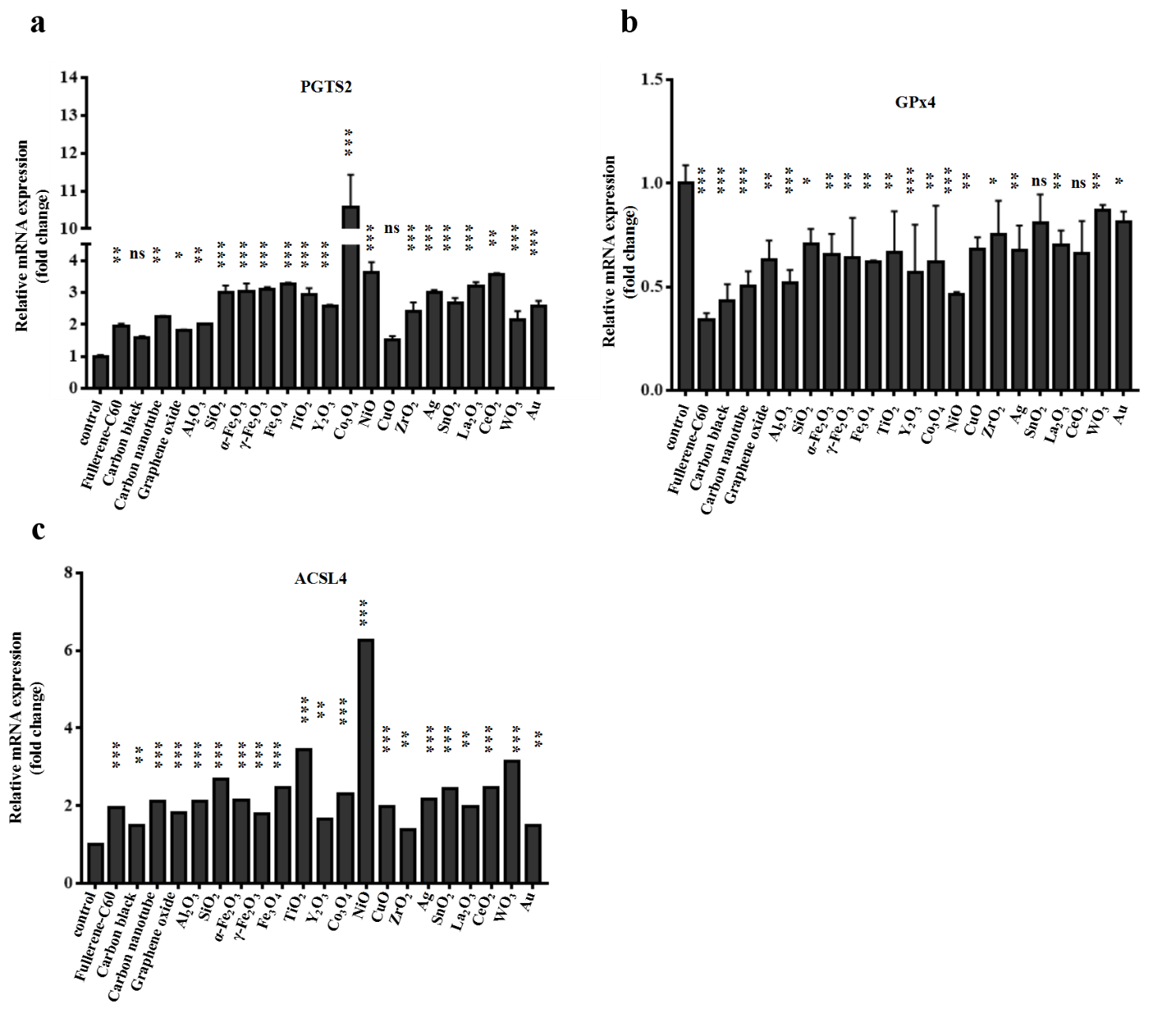


### Supplementary Fig. 21.

ZnO NPs cause intracellular Fe^2+^ elevation in various cell lines. Cells were exposed to 10 μg/mL ZnO NPs for 24 h, and subjected to Lillie divalent iron staining. Red circled images indicated insignificant staining. Scale bar = 20 μm.

**
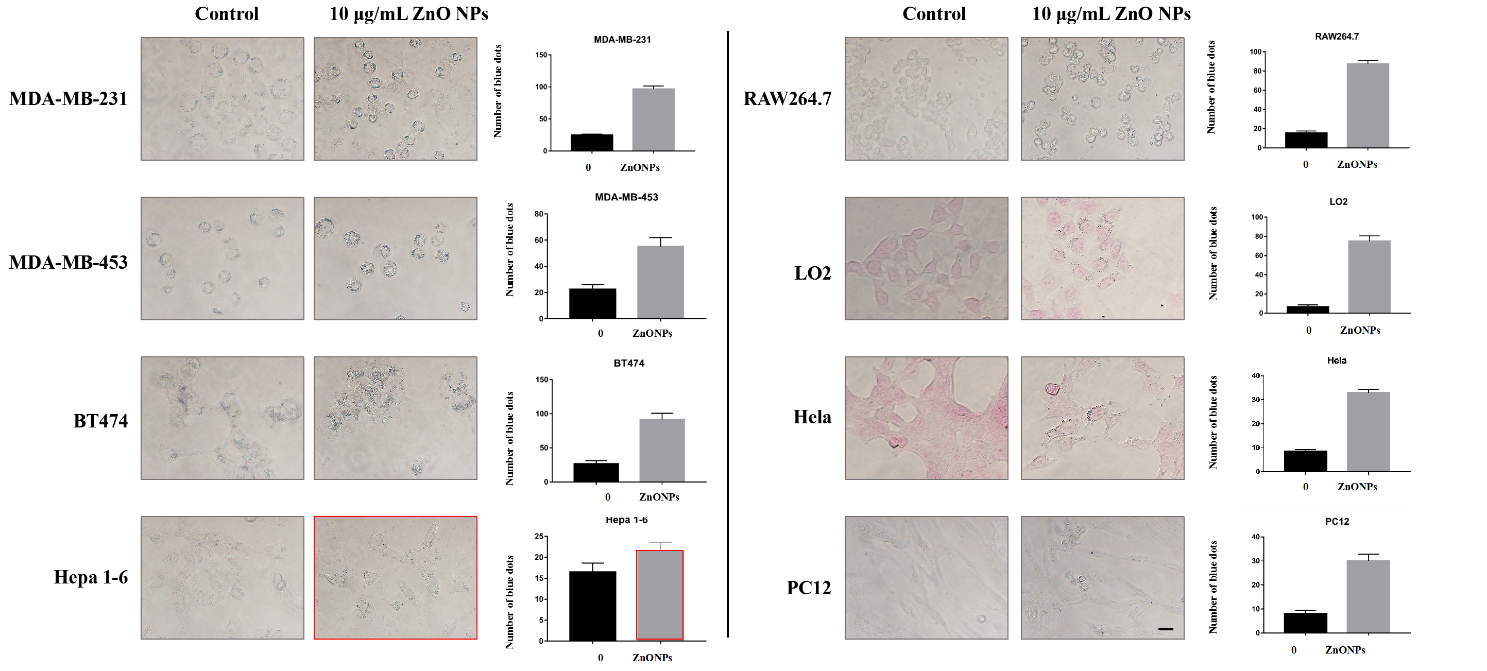
**

### Supplementary Table 1.

Physical Characterization of ZnO and F-ZnO NPs.

| Nanoparticle | Hydrodynamic Size (nm) | | PDI | | Zeta potential (mV) | |
| --- | --- | --- | --- | --- | --- | --- |
|  | H_2_O | 1640 | H_2_O | 1640 | H_2_O | 1640 |
| ZnO NPs | 341±5.1 | 388±7.0 | 0.10±0.04 | 0.27±0.04 | -17.0±0.4 | -8.0 ±0.4 |
| F-ZnO NPs | 380±5.6 | 392±2.1 | 0.11±0.05 | 0.20±0.01 | -18.6±0.1 | -10.0±0.3 |

### Supplementary Table 2.

Characterization of used 21 nanomaterials.

| Nanomaterials | Primary particle size | producer | H_2_O | | RPMI-1640 | |
| --- | --- | --- | --- | --- | --- | --- |
|  |  |  | Zeta (mV) | Size (nm) | Zeta (mV) | Size (nm) |
| C60 |  | Aladdin | 5.4±0.6 | 454.8±32.2 | -10.6±0.4 | 291.5±11.8 |
| Carbon black |  | Macklin | 13.7±2 | 336.1±0 | -9.5±1 | 185.3±37 |
| Carbon nanotube | diameter 20-40 nm, Length 5-15 μm | Shenzhen Nanotech Port Co | -10.6±0.2 | 124.7±10.3 | -10.6±0.4 | 343±9.8 |
| Graphene oxide | Size 0.5-3 μm, thickness 0.55-1.2 nm | Aladdin | -26.7±1.3 | 85.8±47.7 | -11.3±1 | 207.4±39.1 |
| Al_2_O_3_ | 30 nm | Macklin | 10.4±0.3 | 295.1±37.2 | -8.3±0.5 | 503.3±18.2 |
| SiO_2_ | 5-15 nm | sigma | -17.7±0.6 | 199.4±19.1 | -9.9±0.4 | 164.7±21.3 |
| α-Fe_2_O_3_ | 30 nm | Aladdin | -16.5±0.8 | 158.9±9.6 | -10.4±0.3 | 68.7±4.9 |
| γ-Fe_2_O_3_ | 20 nm | Macklin | -14.6±0.9 | 102.5±24.4 | -9.4±0.7 | 140.3±24.9 |
| Fe_3_O_4_ | 20 nm | Meryer | -7.2±0.4 | 841.9±160.4 | -10.5±0.4 | 112.2±15.3 |
| TiO_2_ | 25 nm | Macklin | -26.8±0.6 | 193.8±100.5 | -9.6±1 | 321.4±127.2 |
| Co_3_O_4_ | 30 nm | Macklin | -12.6±0.6 | 284.3±44.4 | -9.9±0.4 | 112.9±16.3 |
| NiO | 30 nm | Aladdin | 32.9±0.1 | 120.6±35.9 | -11.2±1.3 | 161.9±25 |
| CuO | 100-200 nm | Macklin | -15.8±0.4 | 114.6±9.3 | -9.2±0.5 | 278.2±30.5 |
| Y_2_O_3_ | 40 nm | Aladdin | 7.2±0.4 | 304.7±184.4 | -10.3±0.7 | 468.6±0 |
| ZrO_2_ | 50 nm | Macklin | -23.4±0.1 | 159.9±5.6 | -9.6±1 | 246.8±14.9 |
| Ag | 60-120 nm | Macklin | -15.6±0.3 | 67.1±16.5 | -9.5±0.1 | 248.8±17.6 |
| SnO_2_ | 50-70 nm | Macklin | -5.7±0.3 | 249.5±23.5 | -9.6±0.7 | 266.1±0 |
| La_2_O_3_ | 50 nm | Macklin | 10.5±0.6 | 612.1±23.2 | -10.6±0.4 | 291.5±11.8 |
| CeO_2_ | 20-50 nm | Aladdin | -4.8±0.1 | 146.2±46.3 | -9.9±0.8 | 128.2±60.6 |
| WO_3_ | <100 nm | Macklin | -23.4±0.2 | 139±43.2 | -9.9±0.3 | 179.2±78.5 |
| Au | 5-10 nm | Laboratory synthesis | -27.8±1 | 5.7±0.9 | -23.4±0.1 | 117.4±0 |

### Supplementary Table 3.

The sense-strand sequences of siRNA duplexes were used as follows:

| P53 | 5′-CCACCAUCCACUACAACUA-3′ |
| --- | --- |
| SAT1 | 5′-GGAGCUGGCUAAAUAUGAA-3′ |
| ACSL4 | 5′-GACCGAAGGACACAUAUAU-3′ |
| ALOX15 | 5′-GUCGAGAGAUCACUGAAAU-3′ |
| Scrambled | 5′-UUCUCCGAACGUGUCACGU-3′ |

### Supplementary Table 4.

Sequence of all primers used in RT-qPCR experiment.

| **primers** | **Forward** | **Reverse** |
| --- | --- | --- |
| ALOX15 | 5′-GGGCAAGGAGACAGAACTCAA-3′ | 5′-CAGCGGTAACAAGGGAACCT-3′ |
| ACSL4 | 5′-ACTGGCCGACCTAAGGGAG-3′ | 5′-GCCAAAGGCAAGTAGCCAATA-3′ |
| PTGS2 | 5′-ATGCTGACTATGGCTACAAAAGC-3′ | 5′-TCGGGCAATCATCAGGCAC-3′ |
| p53 | 5′-CAGCACATGACGGAGGTTGT-3′ | 5′-TACTCCAAATACTCCACACGC-3′ |
| GPX4 | 5′-GAGGCAAGACCGAAGTAAACTAC-3′ | 5′-CCGAACTGGTTACACGGGAA-3′ |
| SLC7A11 | 5′-TCTCCAAAGGAGGTTACCTGC-3′ | 5′-AGACTCCCCTCAGTAAAGTGAC-3′ |
| SAT1 | 5′-ACCCGTGGATTGGCAAGTTAT-3′ | 5′-TGCAACCTGGCTTAGATTCTTC-3′ |
| DMT1 | 5′-ACTGGCTCAGACATGCAAGAA-3′ | 5′-TTCCGCAAGCCATATTTGTCC-3′ |
| FTL | 5′-CAGCCTGGTCAATTTGTACCT-3′ | 5′-GCCAATTCGCGGAAGAAGTG-3′ |
| FTH | 5′-CGAGGTGGCCGAATCTTCC-3′ | 5′-GTTTGTGCAGTTCCAGTAGTGA-3′ |
| TFRC | 5′-GGCTACTTGGGCTATTGTAAAGG-3′ | 5′-CAGTTTCTCCGACAACTTTCTCT-3′ |
| FPN1 | 5′-CTACTTGGGGAGATCGGATGT-3′ | 5′-CTGGGCCACTTTAAGTCTAGC-3′ |
| BACH1 | 5′-AGTGTAAACTCCGCAGGTATCA-3′ | 5′-TTTGGGGCATAAAGAAGGCAA-3′ |
| MZF1 | 5′-TTCCGGTGCTTCCGCTATG-3′ | 5′-CTCCTTGGAGCGTACCTCT-3′ |
| OPA1 | 5′-TGTGAGGTCTGCCAGTCTTTA-3′ | 5′-TGTCCTTAATTGGGGTCGTTG-3′ |
| MFN1 | 5′-GAGGTGCTATCTCGGAGACAA-3′ | 5′-GCCAATCCCACTAGGGAGAAC-3′ |
| DRP1 | 5′-TTTGACACTTGTGGATTTGCCA-3′ | 5′-AGTGACAGCGAGGATAATGGA -3′ |
| β-actin | 5′-CATGTACGTTGCTATCCAGGC-3′ | 5′-CTACTTATTGTCACGCACGAT-3′ |
